# Supplementary material for: AAK1 activation-mediated iron trafficking drives ferroptotic cell death
Source: Nat Commun. 2025 Dec 17;17:819. doi: 10.1038/s41467-025-67523-9 (PMC12824188; doi:10.1038/s41467-025-67523-9)
Supplement: Supplementary file 1 — Supplementary Information [file 41467_2025_67523_MOESM1_ESM.pdf]

Supplementary Information

AAK1 activation-mediated iron trafficking drives ferroptotic cell death

Li et al.

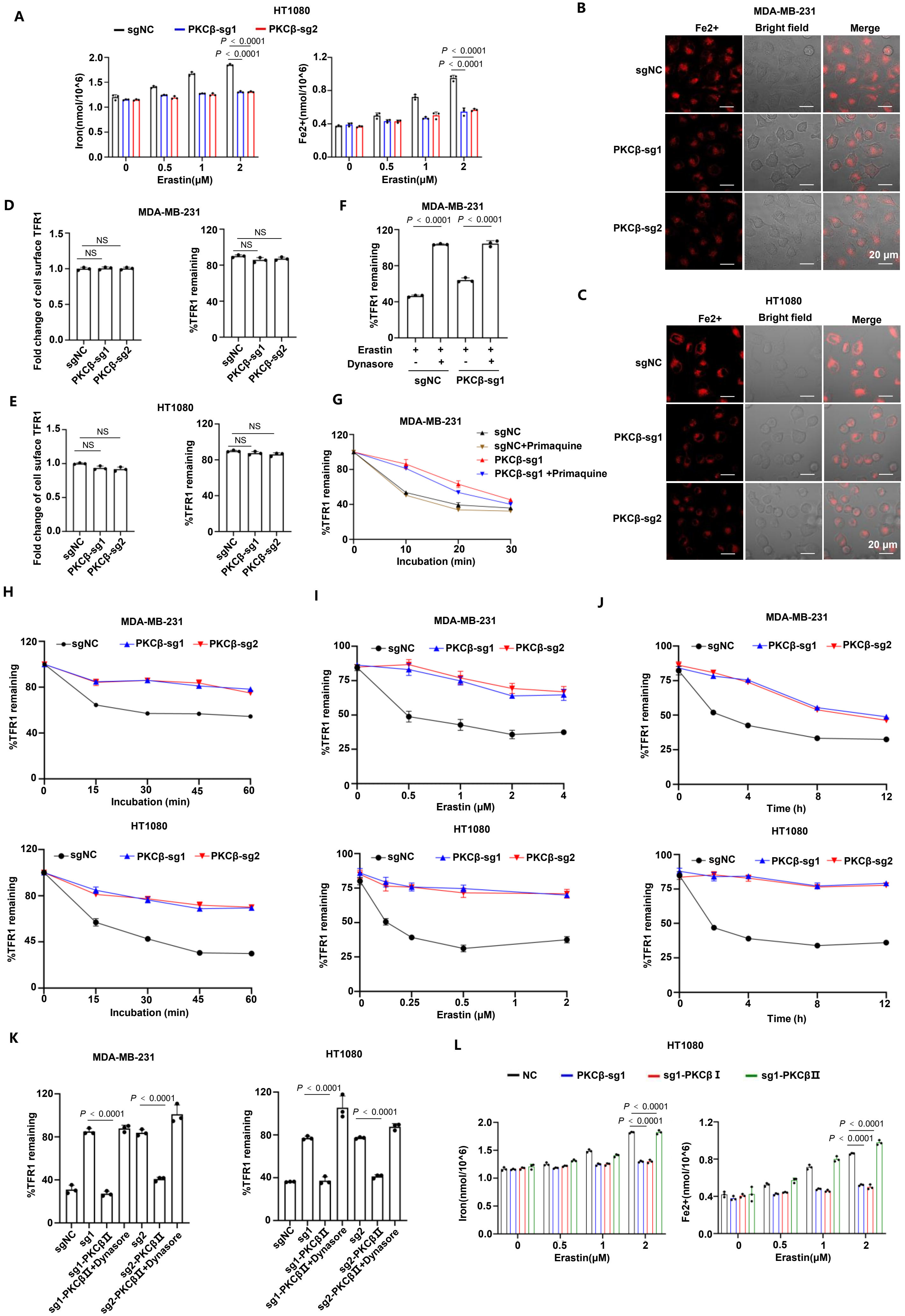

A
HT1080
sgNC
PKCβ-sg1
PKCβ-sg2
P < 0.0001
Iron(nmol/10^6)
Fe2+(nmol/10^6)
Erastin(μM)
B
MDA-MB-231
Fe2+
Bright field
Merge
sgNC
PKCβ-sg1
PKCβ-sg2
20 μm
C
HT1080
D
MDA-MB-231
Fold change of cell surface TFR1
%TFR1 remaining
NS
E
HT1080
Fold change of cell suface TFR1
F
MDA-MB-231
Erastin
Dynasore
G
sgNC+Primaquine
PKCβ-sg1 +Primaquine
Incubation (min)
H
I
Erastin (μM)
J
Time (h)
K
sg1
sg1-PKCβII
sg1-PKCβII+Dynasore
sg2
sg2-PKCβII
sg2-PKCβII+Dynasore
L
NC
sg1-PKCβ I
sg1-PKCβII

Supplementary Fig. 1 PKCβII facilitates the endocytosis of TFR1 and iron uptake in the induction of ferroptosis. **A**, Total cellular iron levels (left) and divalent iron levels (right) were assayed in the indicated HT1080 cells treated with erastin at different concentrations for 12h. **B, C**, Cellular divalent iron levels were assayed in the indicated MDA-MB-231 (**B**) and HT1080 (**C**) cells treated with 4μM or 2μM erastin for 12h respectively. Immunofluorescent staining was used with FerroOrange, a Fe^{2+}-sensitive probe. Images are representative of n = 3 biologically independent experiments. **D, E**, Levels of TFR1 in the cell surface and endocytosis assays were performed in the indicated MDA-MB-231 (**D**) and HT1080 (**E**) cells under resting condition. **F**, Endocytosis assays of TFR1 in the indicated MDA-MB-231 cells treated with 4μM erastin for 12h. Dynasore, 150μM for 1h. **G**, Recycling assays of TFR1 in the indicated MDA-MB-231 cells treated with 4μM erastin for 12h, followed by treatment at 37°C for different incubation time. Primaquine was treated with 200μM during incubation. **H**, Endocytosis assays of TFR1 in the indicated MDA-MB-231 (top) and HT1080 (bottom) cells treated at 37°C for different incubation time after treatment with 4μM or 2μM erastin for 12h respectively. **I**, Endocytosis assays of TFR1 in the indicated MDA-MB-231 (top) and HT1080 (bottom) cells treated with erastin at different concentrations for 12h. **J**, Endocytosis assays of TFR1 in the indicated MDA-MB-231 (top) and HT1080 (bottom) cells treated with 2μM or 1μM erastin respectively for different time periods. **K**, Endocytosis assays of TFR1 in the indicated MDA-MB-231 (left) and HT1080 (right) cells treated with 2μM or 1μM erastin for 12h respectively. dynasore, 150μM for 1h. **L**, Total cellular iron levels (left) and divalent iron levels (right) were assayed in the indicated HT1080 cells treated with erastin at different concentrations for 12h. **A, D, E, L**, Data are presented as means ± SD, n = 3 biologically independent experiments, one-way ANOVA test. **F, K**, Data are presented as means ± SD, n = 3 biologically independent experiments, unpaired two-tailed Student's t test.

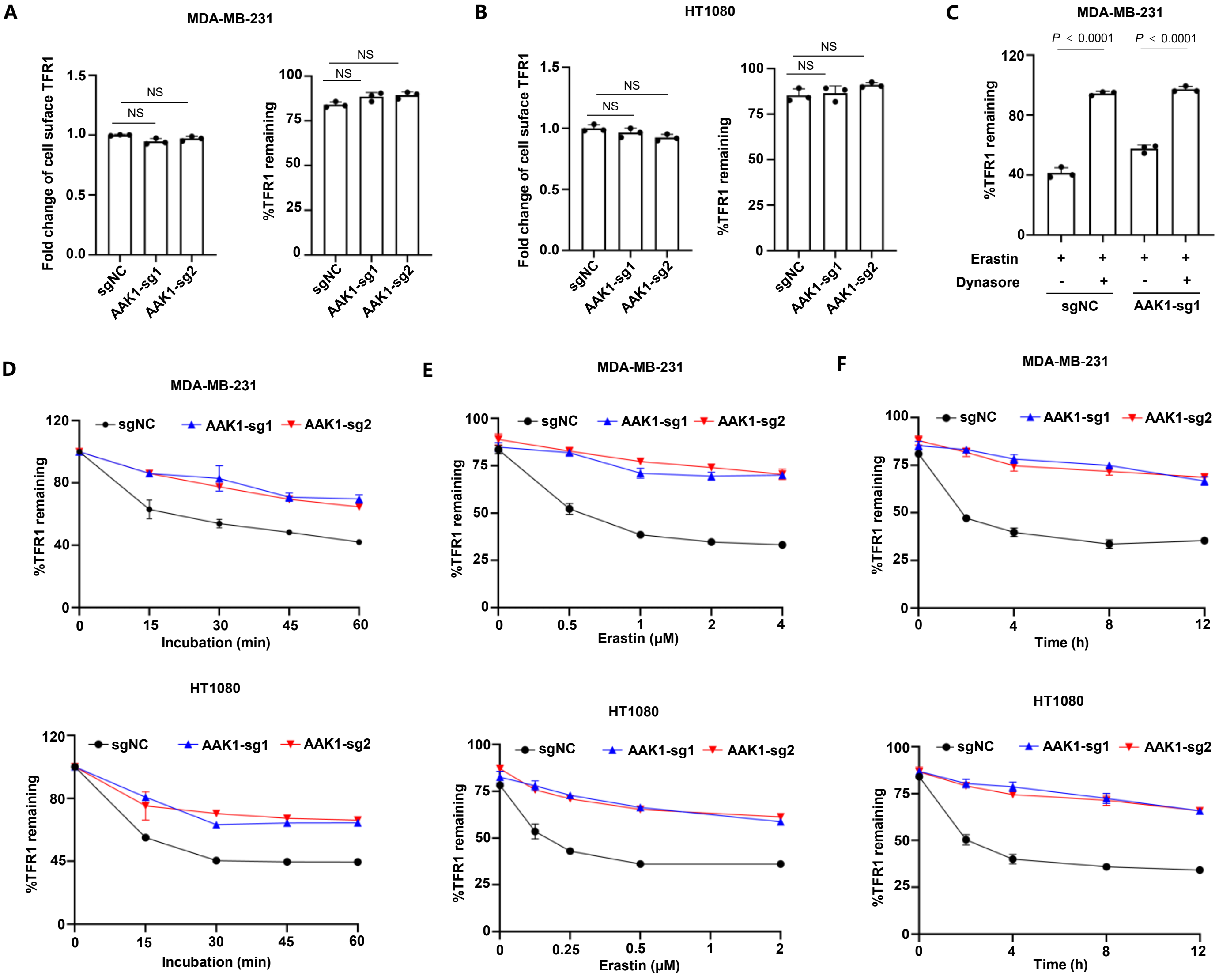

Supplementary Fig. 2 AAK1 facilitates the endocytosis of TFR1 in the induction of ferroptosis. A, B, Levels of TFR1 in the cell surface and endocytosis assays were performed in the indicated MDA-MB-231 (**A**) and HT1080 (**B**) cells under resting-state condition. **C**, Endocytosis assays of TFR1 in the indicated MDA-MB-231 cells treated with 4µM erastin for 12h. Dynasore, 150µM for 1h. **D**, Endocytosis assays of TFR1 in the indicated MDA-MB-231 (top) and HT1080 (bottom) cells treated at 37°C for different incubation time after treatment with 4µM or 2µM erastin for 12h respectively. **E**, Endocytosis assays of TFR1 in the indicated MDA-MB-231 (top) and HT1080 (bottom) cells treated with erastin at different concentrations for 12h. **F**, Endocytosis assays of TFR1 in the indicated MDA-MB-231 (top) and HT1080 (bottom) cells treated with 2µM or 1µM erastin respectively for different time periods. **A, B**, Data are presented as means ± SD, n = 3 biologically independent experiments, one-way ANOVA test. **C**, Data are presented as means ± SD, n = 3 biologically independent experiments, unpaired two-tailed Student's t test.

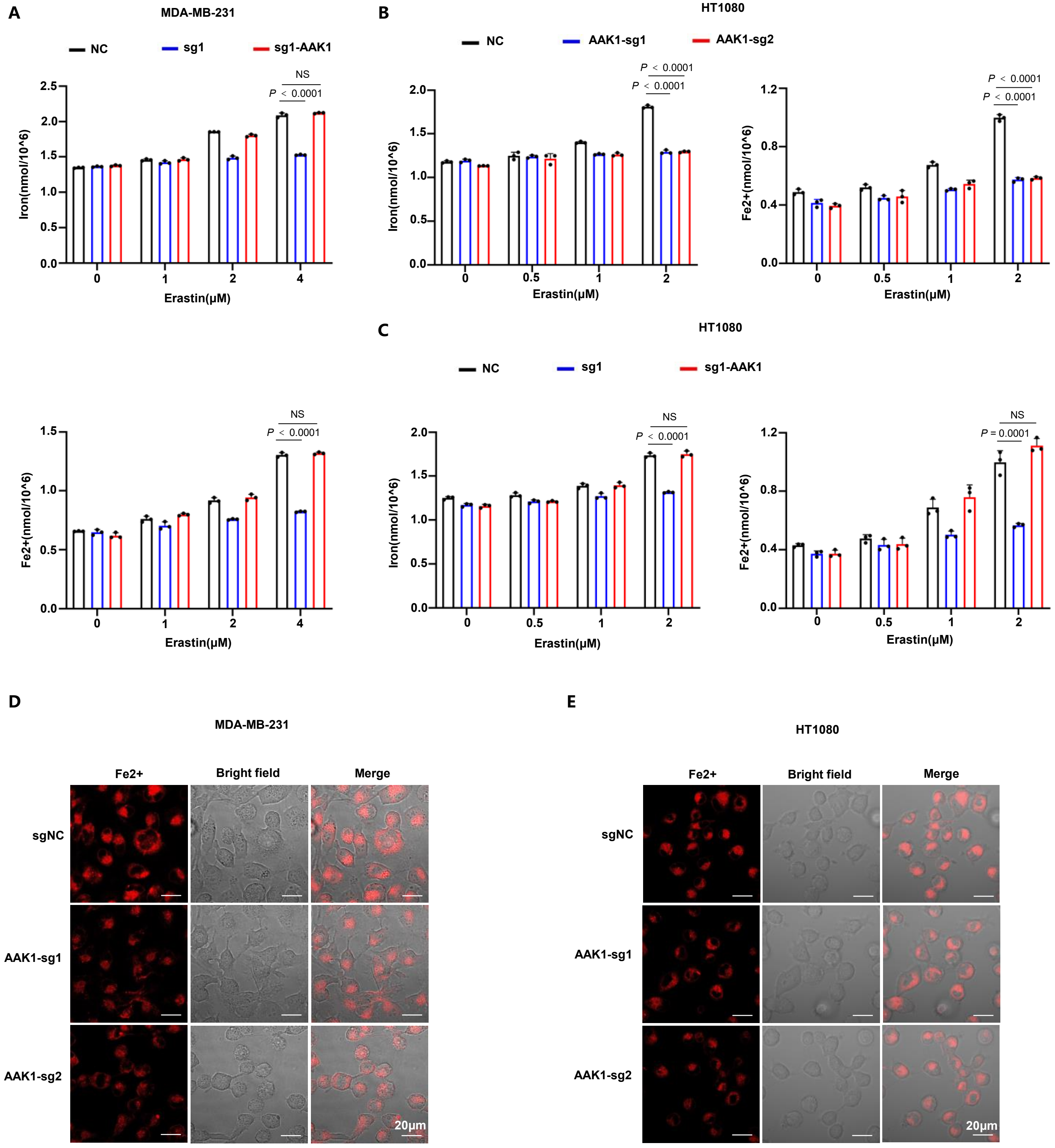

Supplementary Fig. 3 AAK1 promotes iron uptake during ferroptosis. **A**, Total cellular iron levels (top) and divalent iron levels (bottom) were assayed in the indicated MDA-MB-231 cells treated with erastin at different concentrations for 12h. **B, C**, Total cellular iron levels (left) and divalent iron levels (right) were assayed in the indicated HT1080 cells treated with erastin at different concentrations for 12h. **D, E**, Cellular divalent iron levels were assayed in the indicated MDA-MB-231 (**D**) and HT1080 (**E**) cells treated with 4μM or 2μM erastin for 12h respectively. Immunofluorescent staining was used with FerroOrange, a Fe^{2+}-sensitive probe. Images are representative of n = 3 biologically independent experiments. **A-C**, Data are presented as means ± SD, n = 3 biologically independent experiments, one-way ANOVA test.

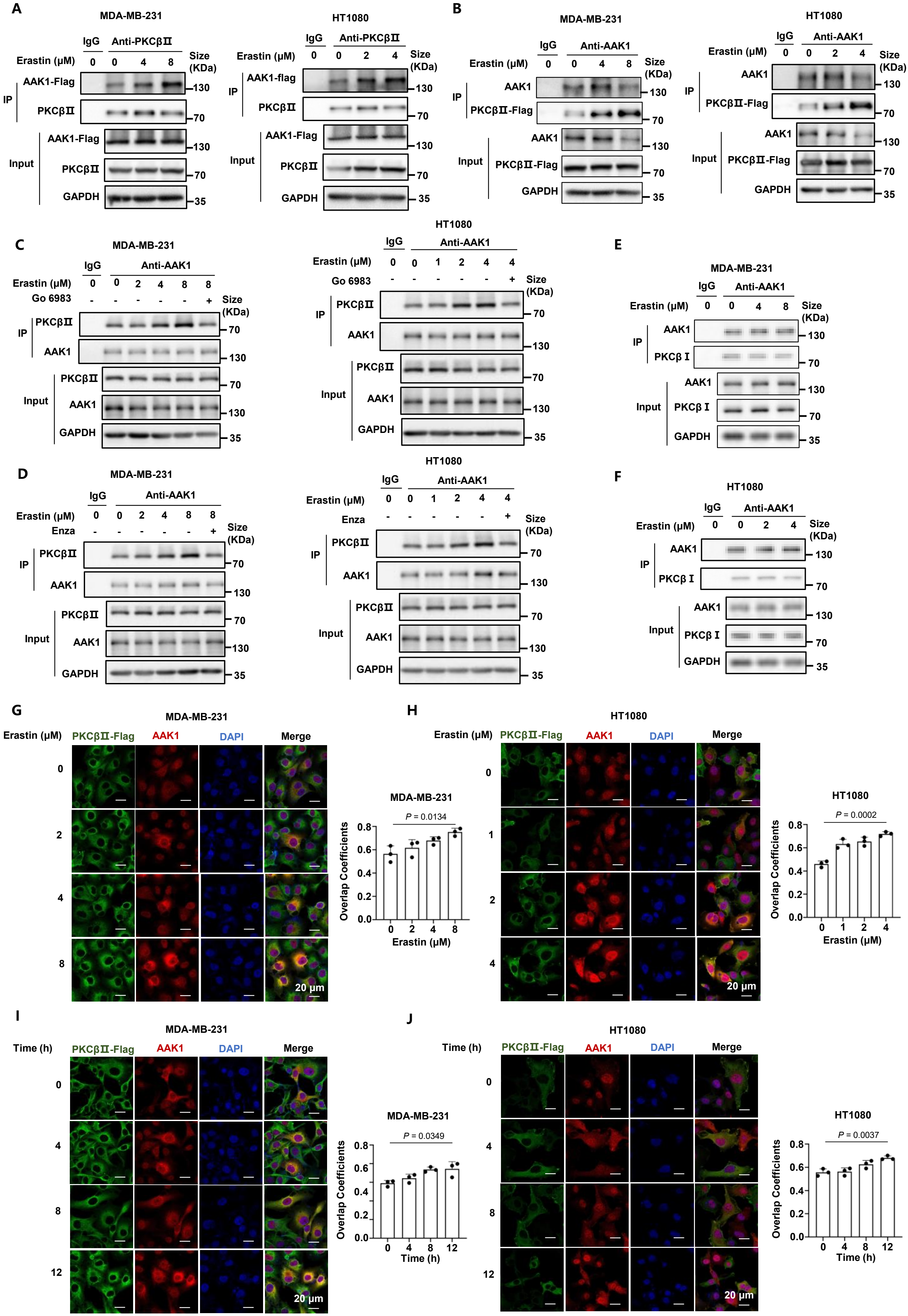
A
MDA-MB-231
IgG
Anti-PKCβⅡ
Erastin (μM)
0
0
4
8
Size (KDa)
IP
AAK1-Flag
PKCβⅡ
Input
AAK1-Flag
PKCβⅡ
GAPDH
130
70
130
70
35
HT1080
IgG
Anti-PKCβⅡ
Erastin (μM)
0
0
2
4
Size (KDa)
IP
AAK1-flag
PKCβⅡ
Input
AAK1-Flag
PKCβⅡ
GAPDH
130
70
130
70
35
B
MDA-MB-231
IgG
Anti-AAK1
Erastin (μM)
0
0
4
8
Size (KDa)
IP
AAK1
PKCβⅡ-Flag
Input
AAK1
PKCβⅡ-Flag
GAPDH
130
70
130
70
35
HT1080
IgG
Anti-AAK1
Erastin (μM)
0
0
2
4
Size (KDa)
IP
AAK1
PKCβⅡ-Flag
Input
AAK1
PKCβⅡ-Flag
GAPDH
130
70
130
70
35
C
MDA-MB-231
IgG
Anti-AAK1
Erastin (μM)
0
0
2
4
8
8
Go 6983
- - - - - +
Size (KDa)
IP
PKCβⅡ
AAK1
Input
PKCβⅡ
AAK1
GAPDH
70
130
70
130
35
HT1080
IgG
Anti-AAK1
Erastin (μM)
0
0
1
2
4
4
Go 6983
- - - - - +
Size (KDa)
IP
PKCβⅡ
AAK1
Input
PKCβⅡ
AAK1
GAPDH
70
130
70
130
35
E
MDA-MB-231
IgG
Anti-AAK1
Erastin (μM)
0
0
4
8
Size (KDa)
IP
AAK1
PKCβⅠ
Input
AAK1
PKCβⅠ
GAPDH
130
70
130
70
35
D
MDA-MB-231
IgG
Anti-AAK1
Erastin (μM)
0
0
2
4
8
8
Enza
- - - - - +
Size (KDa)
IP
PKCβⅡ
AAK1
Input
PKCβⅡ
AAK1
GAPDH
70
130
70
130
35
HT1080
IgG
Anti-AAK1
Erastin (μM)
0
0
1
2
4
4
Enza
- - - - - +
Size (KDa)
IP
PKCβⅡ
AAK1
Input
PKCβⅡ
AAK1
GAPDH
70
130
70
130
35
F
HT1080
IgG
Anti-AAK1
Erastin (μM)
0
0
2
4
Size (KDa)
IP
AAK1
PKCβⅠ
Input
AAK1
PKCβⅠ
GAPDH
130
70
130
70
35
G
MDA-MB-231
Erastin (μM)
PKCβⅡ-Flag
AAK1
DAPI
Merge
0
2
4
8
20 μm
MDA-MB-231
P = 0.0134
Overlap Coefficients
0.0
0.2
0.4
0.6
0.8
0
2
4
8
Erastin (μM)
H
HT1080
Erastin (μM)
PKCβⅡ-Flag
AAK1
DAPI
Merge
0
1
2
4
20 μm
HT1080
P = 0.0002
Overlap Coefficients
0.0
0.2
0.4
0.6
0.8
0
1
2
4
Erastin (μM)
I
MDA-MB-231
Time (h)
PKCβⅡ-Flag
AAK1
DAPI
Merge
0
4
8
12
20 μm
MDA-MB-231
P = 0.0349
Overlap Coefficients
0.0
0.2
0.4
0.6
0.8
0
4
8
12
Time (h)
J
HT1080
Time (h)
PKCβⅡ-Flag
AAK1
DAPI
Merge
0
4
8
12
20 μm
HT1080
P = 0.0037
Overlap Coefficients
0.0
0.2
0.4
0.6
0.8
0
4
8
12
Time (h)

Supplementary Fig. 4 PKCβII interacts with and phosphorylates AAK1 during ferroptosis. A, Endogenous PKCβII was immunoprecipitated from MDA-MB-231 (left) and HT1080 (right) cells treated with erastin at different concentrations for 12h, followed by immunoblots using an antibody against AAK1-Flag to establish the interaction of endogenous PKCβII with exogenous AAK1. **B**, Endogenous AAK1 was immunoprecipitated from MDA-MB-231 (left) and HT1080 (right) cells treated with erastin at different concentrations for 12h, followed by immunoblots using an antibody against PKCβII-Flag to establish the interaction of endogenous AAK1 with exogenous PKCβII. **C, D**, Endogenous AAK1 was immunoprecipitated from MDA-MB-231 (left) and HT1080 (right) cells treated with erastin at different concentrations for 12h with/without Go6983 (**C**) or enzastaurin (**D**) followed by immunoblots using a PKCβII-specific antibody. G06983, 5μM; Enza, 5μM enzastaurin. **E, F**, Endogenous AAK1 was immunoprecipitated from MDA-MB-231 (**E**) and HT1080 (**F**) cells treated with erastin at different concentrations for 12h, followed by immunoblots using a PKCβI-specific antibody to establish the interaction of endogenous AAK1 with endogenous PKCβI. **G, H**, The co-localization of PKCβII with AAK1 performed by immunofluorescence in MDA-MB-231 (**G**) and HT1080 (**H**) cells treated with erastin at different concentrations for 12h. **I, J**, The co-localization of PKCβII with AAK1 performed by immunofluorescence in MDA-MB-231 (**I**) and HT1080 (**J**) cells treated with 10μM or 5μM erastin respectively for different time periods. **A-F**, Data are representative of n = 3 biologically independent experiments. **G-J**, Images are representative of n = 3 biologically independent experiments. Data are presented as means ± SD, n = 3 biologically independent experiments, unpaired two-tailed Student's t test.

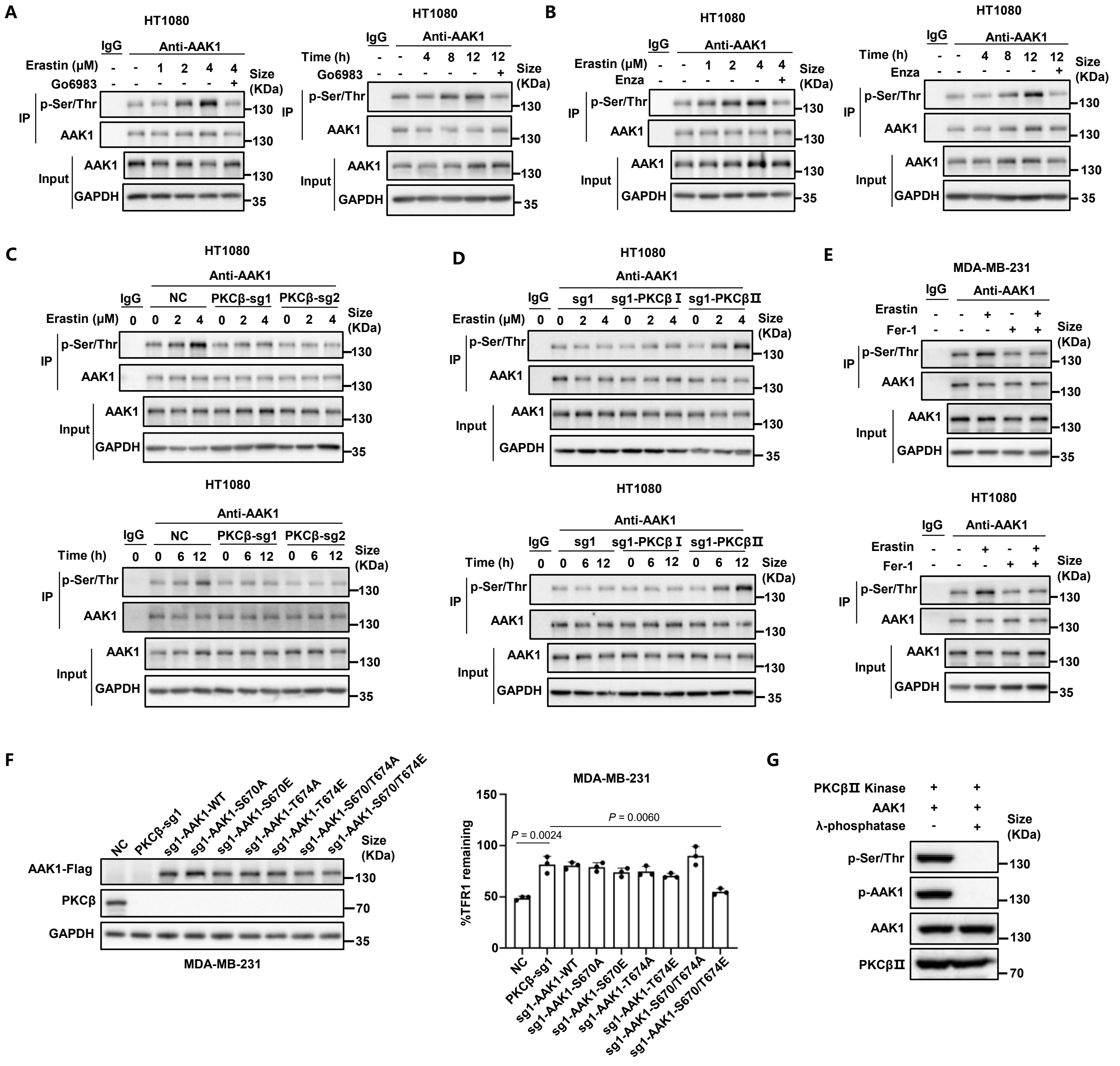

Supplementary Fig. 5 PKCβII-dependent phosphorylation of S670/T674 of AAK1 contributes to the endocytosis of TFR1. **A**, Total phosphorylation levels of AAK1 in HT1080 cells treated with erastin at different concentrations (left) and for different time periods (right) as indicated, with/without Go6983 by Immunoblots. Go6983, 5μM. **B**, Total phosphorylation levels of AAK1 in HT1080 cells treated with erastin at different concentrations (left) and for different time periods (right) as indicated, with/without enzastaurin by Immunoblots. Enza, 5μM enzastaurin. **C**, Total phosphorylation levels of AAK1 in the indicated HT1080 cells treated with erastin at different concentrations (top) and for different time periods (bottom) by Immunoblots. **D**, Total phosphorylation levels of AAK1 in the indicated HT1080 cells treated with erastin at different concentrations (top) and for different time periods (bottom) by Immunoblots. **E**, Total phosphorylation levels of AAK1 in MDA-MB-231 (top) and HT1080 (bottom) cells treated with/without 10μM or 5μM erastin for 12h respectively and with/without 10μM Fer-1 for 12h by Immunoblots. **F**, *PKCβ*-knockout MDA-MB-231 cells were transfected into wild type or various mutant *AAK1* plasmids and verified by immunoblots. Endocytosis assays of TFR1 in the indicated cells treated with 2μM erastin for 12h. **G**, Purified AAK1 proteins were incubated with recombinant activated PKCβII kinase with/without λ-phosphatase for 0.5h in kinase buffer with ATP in vitro. **A-G**, Data are representative of n = 3 biologically independent experiments. **F**, Data are presented as means ± SD, n = 3 biologically independent experiments, unpaired two-tailed Student's t test.

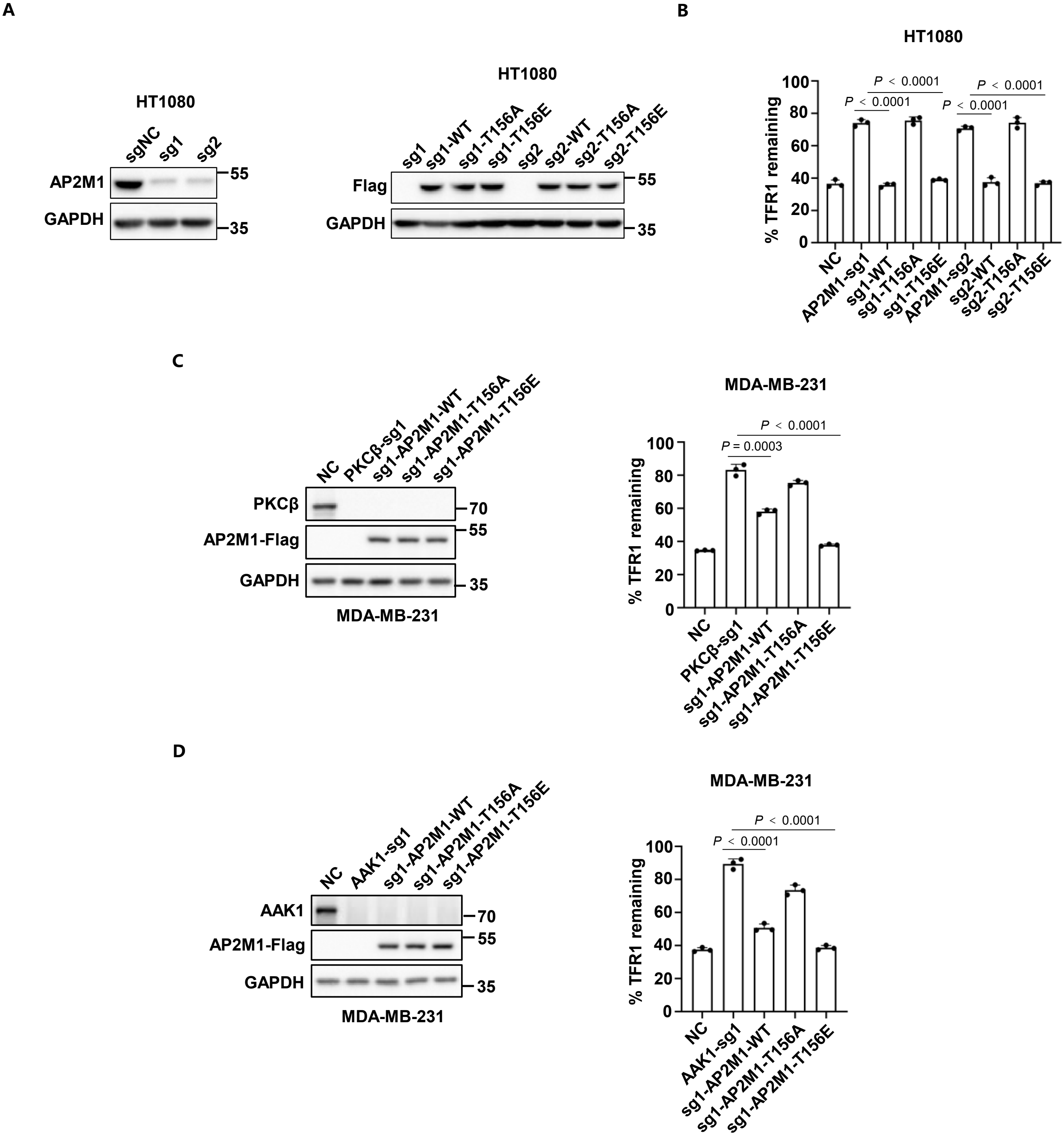

Supplementary Fig. 6 AP2M1 promotes the endocytosis of TFR1 and iron uptake linked to ferroptosis. A, Knockout of *AP2M1* was performed in HT1080 cells using single guide RNAs (sgRNAs). Plasmids of *AP2M1-WT*, *AP2M1-T156A* and *AP2M1-T156E* were transfected into AP2M1-knockout cells. These cells were verified by immunoblots. **B**, Endocytosis assays of TFR1 in the indicated HT1080 cells treated with 1μM erastin for 12h. **C**, *PKCβ*-knockout MDA-MB-231 cells were transfected with plasmids of *AP2M1-WT*, *AP2M1-T156A* or *AP2M1-T156E* and verified by immunoblots. Endocytosis assays of TFR1 were performed in these cells treated with 2μM erastin for 12h. **D**, *AAK1*-knockout MDA-MB-231 cells were transfected with plasmids of *AP2M1-WT*, *AP2M1-T156A* or *AP2M1-T156E* and verified by immunoblots. Endocytosis assays of TFR1 were performed in these cells treated with 2μM erastin for 12h. **A, C, D**, Data are representative of n = 3 biologically independent experiments. **B-D**, Data are presented as means ± SD, n = 3 biologically independent experiments, unpaired two-tailed Student's t test.

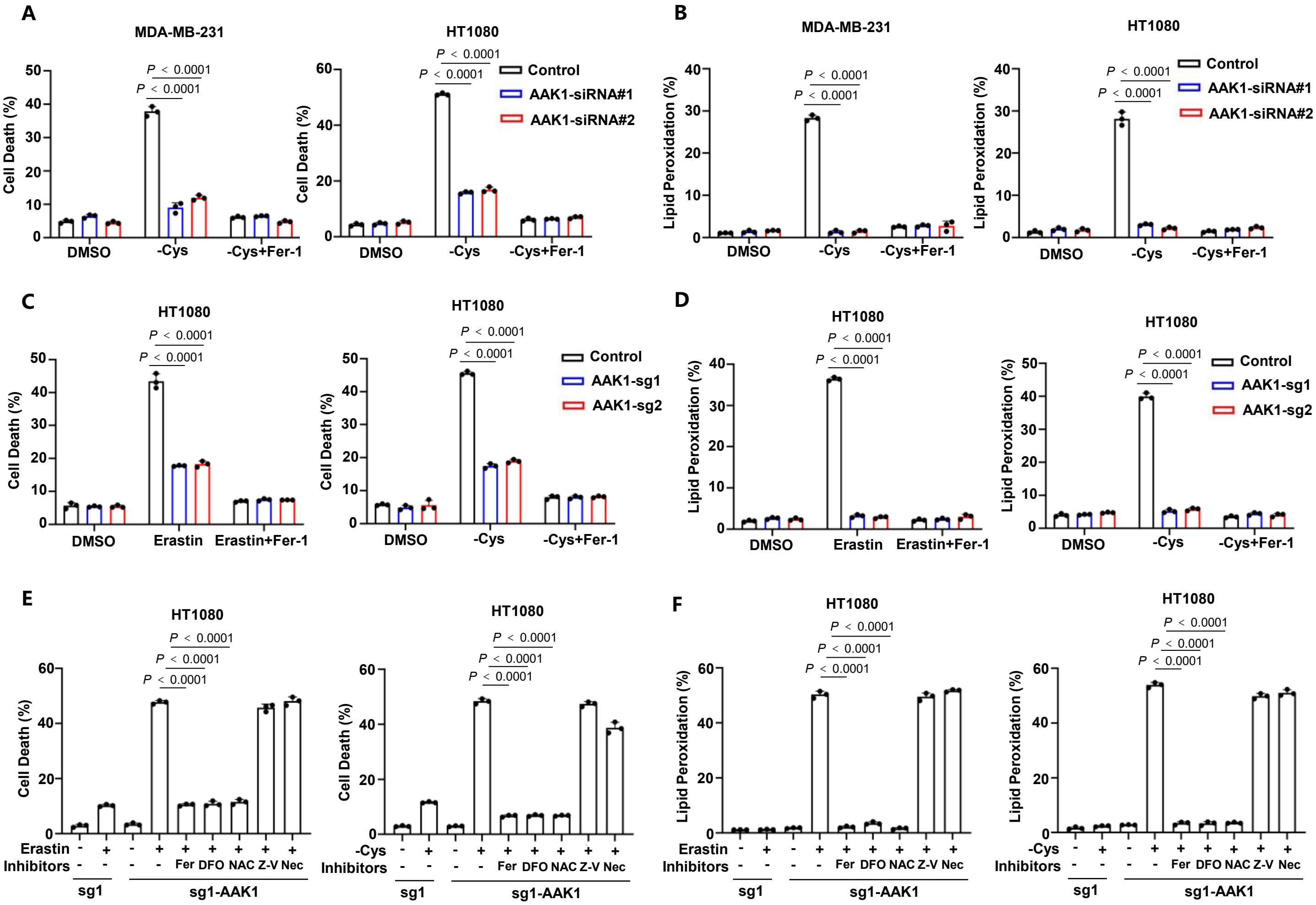

Supplementary Fig. 7 Genetic depletion of AAK1 inhibits ferroptosis. A, B, Cell-death (**A**) and lipid-peroxidation (**B**) measurements for the indicated MDA-MB-231 (left) and HT1080 (right) cells treated with cystine deprivation with/without 10μM Fer-1 for 12h. -Cys, cystine deprivation. **C, D**, Cell-death (**C**) and lipid-peroxidation (**D**) measurements for the indicated HT1080 cells treated with erastin (left) or cystine deprivation (right) with/without Fer-1 for 12h. erastin, 5μM; -Cys, cystine deprivation; Fer-1, 5μM. **E, F**, Cell-death (**E**) and lipid-peroxidation (**F**) measurements for the indicated HT1080 cells treated with erastin (left) or cystine deprivation (right) with/without various cell death inhibitors for 12h. erastin, Fer-1 and DFO, 5μM; -Cys, cystine deprivation; NAC, 5mM N-acetyl-cysteine; Z-V, 10μM Z-VAD-FMK; Nec, 2μM necrostatin-1s. **A-D**, Data are presented as means ± SD, n = 3 biologically independent experiments, one-way ANOVA test. **E, F**, Data are presented as means ± SD, n = 3 biologically independent experiments, unpaired two-tailed Student's t test.

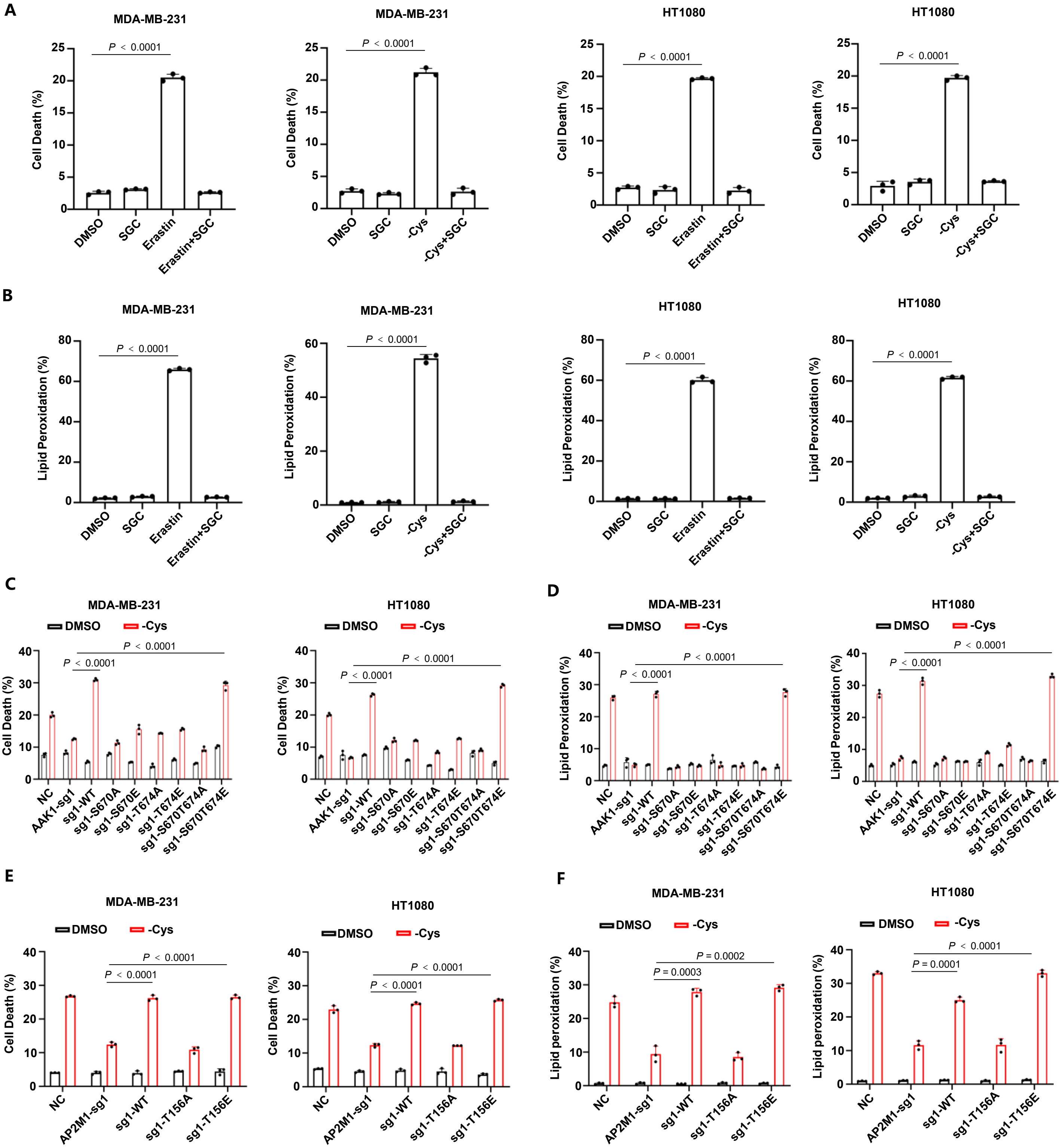

Supplementary Fig. 8 Mutation of AAK1 or AP2M1 inhibits ferroptosis. A, B, Cell-death (**A**) and lipid-peroxidation (**B**) measurements for MDA-MB-231 (left) and HT1080 (right) cells treated with erastin or cystine deprivation with/without SGC-AAK1-1 for 12h. erastin, 10μM for MDA-MB-231 and 5μM for HT1080; -Cys, cystine deprivation; SGC, 10μM SGC-AAK1-1. **C, D**, Cell-death (**C**) and lipid-peroxidation (**D**) measurements for the indicated MDA-MB-231 (left) and HT1080 (right) cells treated with cystine deprivation for 12h. -Cys, cystine deprivation. **E, F**, Cell-death (**E**) and lipid-peroxidation (**F**) measurements for the indicated MDA-MB-231 (left) and HT1080 (right) cells treated with cystine deprivation for 12h. -Cys, cystine deprivation. **A-F**, Data are presented as means ± SD, n = 3 biologically independent experiments, unpaired two-tailed Student's t test.

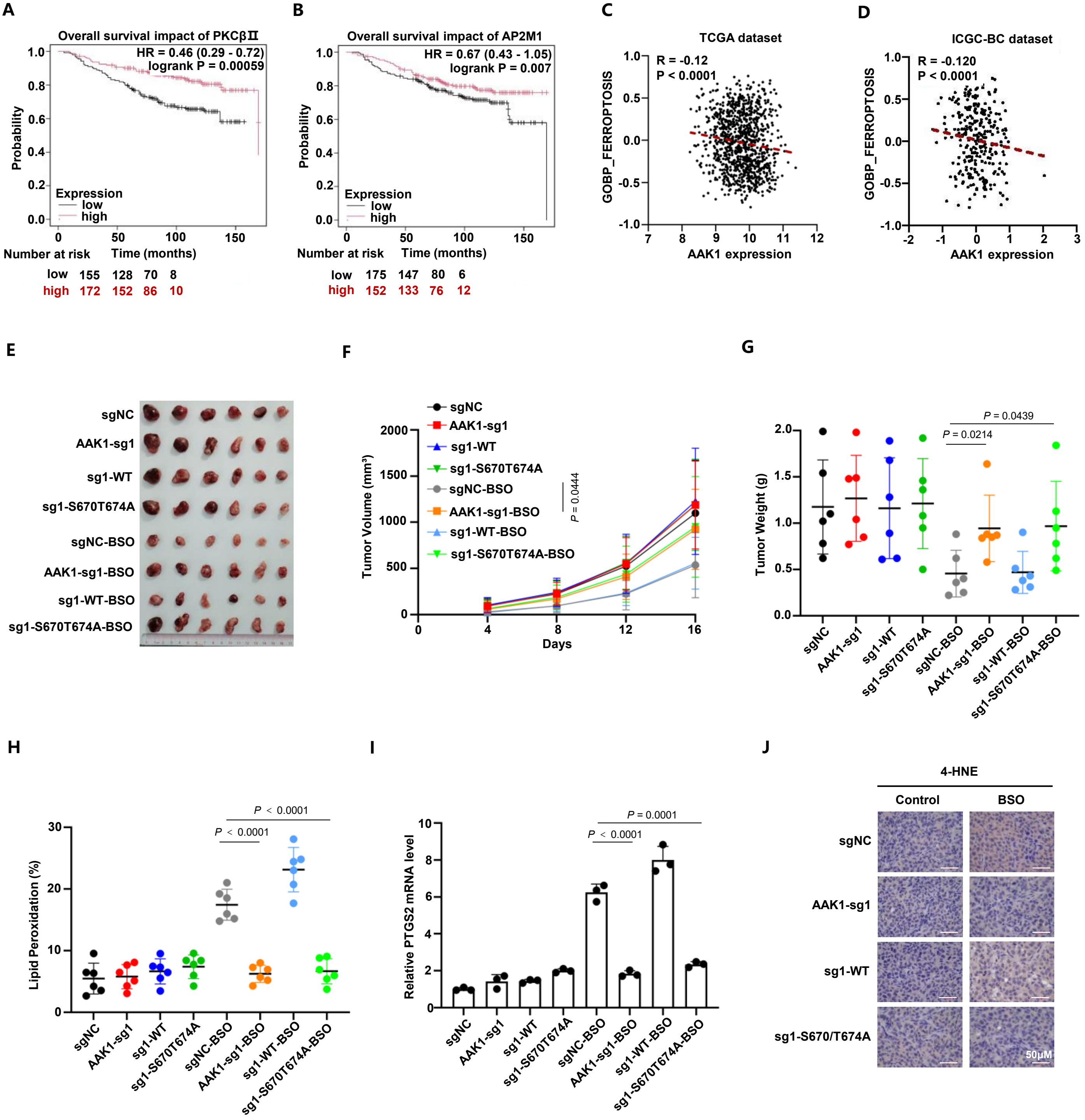

Supplementary Fig. 9 PKCβII-AAK1-AP2M1 pathway inhibits tumor growth through the induction of ferroptosis. A, B, Kaplan–Meier analysis of overall survival related to the expression of *PKCβII* (**A**) and *AP2M1* (**B**) for breast cancer patients. **C, D**, Correlation analysis between the expression of *AAK1* and GOBP gene set containing anti-ferroptosis genes based on TCGA dataset (**C**) and ICGC-BC dataset (**D**). **E-G**, Tumor volume and weight of xenograft tumors formed by the indicated MDA-MB-231 cells treated with DMSO or BSO. **H**, Lipid-peroxidation measurement for tumor cells isolated from the indicated tumors. **I**, Relative mRNA level of *PTGS2* detected by RT-qPCR. These samples were tumor cells isolated from the indicated tumors. **J**, Representative immunohistochemical images of 4-HNE in the indicated tumor tissues. **F,** Data are presented as means ± SD, n = 3 biologically independent experiments, two-way ANOVA test. **G-I**, Data are presented as means ± SD, n = 6 independent samples, unpaired two-tailed Student's t test.

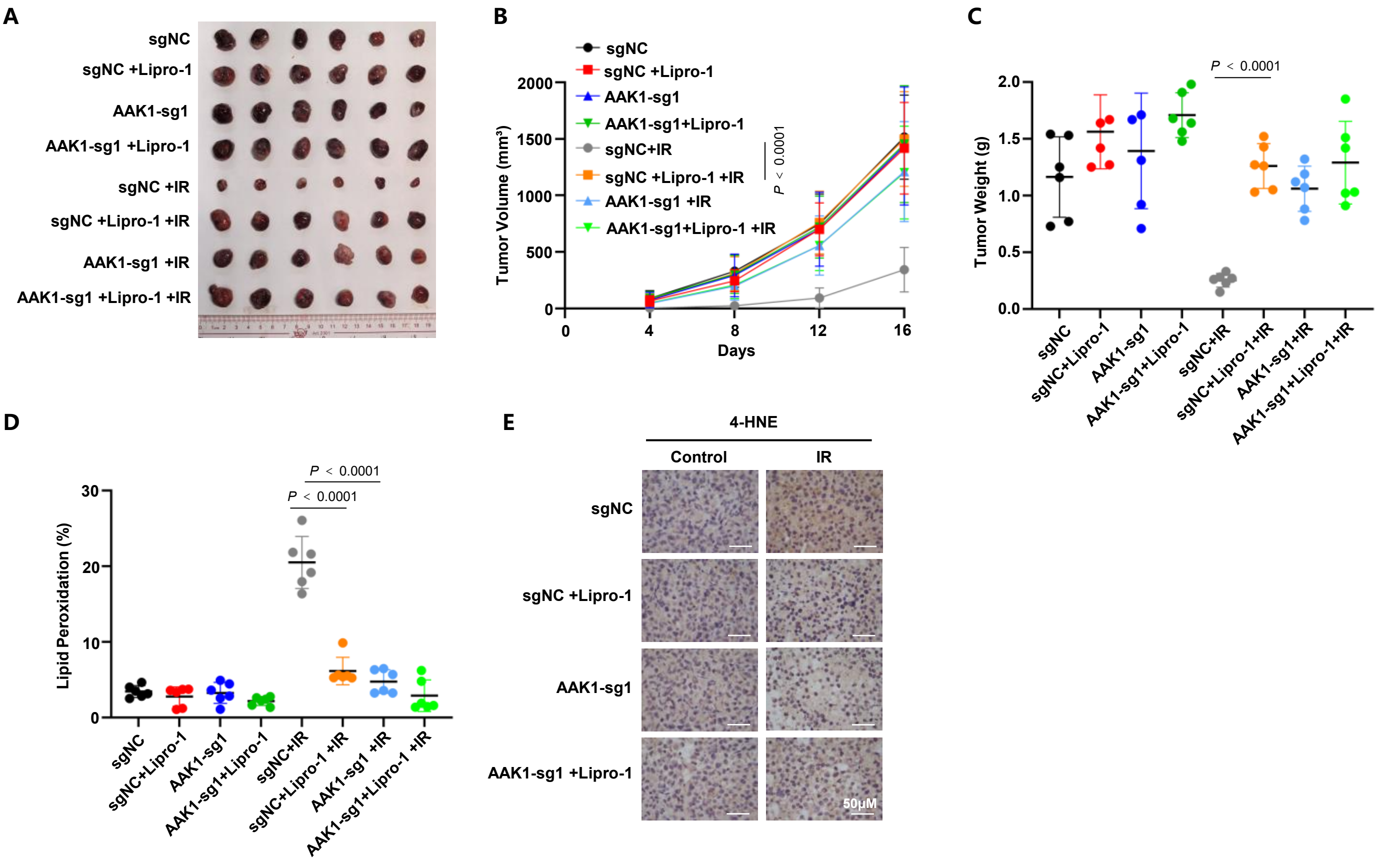

Supplementary Fig. 10 PKCβII-AAK1-AP2M1 pathway enhances the radiosensitivity of tumors to IR. A-C, Tumor volume and weight of xenograft tumors formed by the indicated MDA-MB-231 cells treated with DMSO or Lipro-1, with or without IR. Lipro-1, 10μM. IR, 10Gy. **D**, Lipid-peroxidation measurement for tumor cells isolated from the indicated tumors. **E**, Representative immunohistochemical images of 4-HNE in the indicated tumor tissues. **B,** Data are presented as means ± SD, n = 3 biologically independent experiments, two-way ANOVA test. **C, D**, Data are presented as means ± SD, n = 6 independent samples, unpaired two-tailed Student's t test.
