# Supplementary material for: AAK1 activation-mediated iron trafficking drives ferroptotic cell death
Source: Nat Commun. 2025 Dec 17;17:819. doi: 10.1038/s41467-025-67523-9 (PMC12824188; doi:10.1038/s41467-025-67523-9)
Supplement: Supplementary file 2 — Description of Additional Supplementary Files [file 41467_2025_67523_MOESM2_ESM.pdf]

Description of Additional Supplementary Files

Supplementary Data 1: This file is the raw data of the proteinomic analysis that revealed the changes of phosphorylation levels of proteins in three individual wild type and PKCβ-knockout MDA-MB-231 cells. Potential phosphorylation sites of each protein were included in the file.
