# Supplementary material for: AAK1 activation-mediated iron trafficking drives ferroptotic cell death
Source: Nat Commun. 2025 Dec 17;17:819. doi: 10.1038/s41467-025-67523-9 (PMC12824188; doi:10.1038/s41467-025-67523-9)
Supplement: Supplementary file 4 — Reporting Summary [file 41467_2025_67523_MOESM4_ESM.pdf]

nature portfolio

Corresponding author(s): Xiaofeng Zhu. NCOMMS-25-30255-T

Last updated by author(s): Nov 19, 2025

Reporting Summary

Nature Portfolio wishes to improve the reproducibility of the work that we publish. This form provides structure for consistency and transparency in reporting. For further information on Nature Portfolio policies, see our Editorial Policies and the Editorial Policy Checklist.

Statistics

For all statistical analyses, confirm that the following items are present in the figure legend, table legend, main text, or Methods section.

n/a	Confirmed	
☐	☒	The exact sample size (*n*) for each experimental group/condition, given as a discrete number and unit of measurement
☐	☒	A statement on whether measurements were taken from distinct samples or whether the same sample was measured repeatedly
☐	☒	The statistical test(s) used AND whether they are one- or two-sided<br>*Only common tests should be described solely by name; describe more complex techniques in the Methods section.*
☒	☐	A description of all covariates tested
☐	☒	A description of any assumptions or corrections, such as tests of normality and adjustment for multiple comparisons
☐	☒	A full description of the statistical parameters including central tendency (e.g. means) or other basic estimates (e.g. regression coefficient) AND variation (e.g. standard deviation) or associated estimates of uncertainty (e.g. confidence intervals)
☐	☒	For null hypothesis testing, the test statistic (e.g. *F*, *t*, *r*) with confidence intervals, effect sizes, degrees of freedom and *P* value noted<br>*Give P values as exact values whenever suitable.*
☒	☐	For Bayesian analysis, information on the choice of priors and Markov chain Monte Carlo settings
☒	☐	For hierarchical and complex designs, identification of the appropriate level for tests and full reporting of outcomes
☐	☒	Estimates of effect sizes (e.g. Cohen's *d*, Pearson's *r*), indicating how they were calculated

Our web collection on statistics for biologists contains articles on many of the points above.

Software and code

Policy information about availability of computer code

Data collection	All flow cytometry analyses were conducted on CytoFLEX (Beckman), and the data were analysed using FlowJo software (FlowJo Vx.10.0.7), or CytExpert software (CytExpert 2.4) according to manufacturers' instructions. The confocal microscope (LSM880; Carl Zeiss) was used for immunofluorescence analysis. Quantification of Western Blots was performed using ImageJ software. IHC image acquisition was performed using a KFBIO Digital Pathology Slide Scanner. The relative expression of PKCβII , p-AAK1 and 4-HNE using tissue chips from triple-negative patients was quantified using the pathology image analysis software HALO (Indica Labs). The samples for proteinomics was analyzed by Beijing Omics Biotechnology Co., Ltd. In addition, we acknowledge the use of BioRender (https://biorender.com) for the creation of Figure 1D and Figure 7.
Data analysis	Statistical analyses were conducted using GraphPad Prism 8.0.2 (GraphPad, La Jolla, CA, USA). The quantification of western blots and immunofluorescence was analyzed using ImageJ software. The p values of Kaplan-Meier overall survival curves for TCGA cancer patients were assessed using the two-sided log-rank test. The relationship between the expression of PKCβII and p-AAK1 or 4-HNE was assessed using the two-sided Pearson Chi-square (χ2) test. The results presented as the mean ± SD were analysed by an unpaired Student's t test, or one-way ANOVA test, or two-way ANOVA test using GraphPad Prism 8.0.2. All the statistical tests were two-sided, p < 0.05 was considered statistically significant.

For manuscripts utilizing custom algorithms or software that are central to the research but not yet described in published literature, software must be made available to editors and reviewers. We strongly encourage code deposition in a community repository (e.g. GitHub). See the Nature Portfolio guidelines for submitting code & software for further information.

Data

Policy information about availability of data

All manuscripts must include a data availability statement. This statement should provide the following information, where applicable:

- Accession codes, unique identifiers, or web links for publicly available datasets
- A description of any restrictions on data availability
- For clinical datasets or third party data, please ensure that the statement adheres to our policy

Overall survival and relapse free survival related to the expression of AAK1 for breast cancer patients were performed by Kaplan–Meier analysis (https://kmplot.com/analysis/). The relative expression of AAK1 in primary tumor tissues of breast cancer or normal tissues was analysed by using The Cancer Genome Atlas (TCGA, https://www.cancer.gov/ccg/research/genome-sequencing/tcga). GOBP gene set containing anti-ferroptosis genes was used for the correlation analysis of the expression of AAK1 based on TCGA dataset and ICGC-BC dataset (https://www.gsea-msigdb.org/gsea/msigdb/human/geneset/GOBP_FERROPTOSIS). The raw numbers for graphs are available in the Source Data file. Specific data p values are included in the figures or supplementary information. Unprocessed scans of western blots are available in the Source Data file. The raw data of phosphoproteome which detected the phosphorylation levels of proteins in PKCβ-knockout MDA-MB-231 cell lines during ferroptosis has been supplied as a supplementary data file linked to this paper. All data are included in the Supplementary Information or available from the authors.

Research involving human participants, their data, or biological material

Policy information about studies with human participants or human data. See also policy information about sex, gender (identity/presentation), and sexual orientation and race, ethnicity and racism.

Reporting on sex and gender	N/A,no human research in this manuscript.
Reporting on race, ethnicity, or other socially relevant groupings	N/A,no human research in this manuscript.
Population characteristics	N/A,no human research in this manuscript.
Recruitment	N/A,no human research in this manuscript.
Ethics oversight	N/A,no human research in this manuscript.

Note that full information on the approval of the study protocol must also be provided in the manuscript.

Field-specific reporting

Please select the one below that is the best fit for your research. If you are not sure, read the appropriate sections before making your selection.

☒ Life sciences ☐ Behavioural & social sciences ☐ Ecological, evolutionary & environmental sciences

For a reference copy of the document with all sections, see nature.com/documents/nr-reporting-summary-flat.pdf

Life sciences study design

All studies must disclose on these points even when the disclosure is negative.

Sample size	Sample size was chosen based on the need to have sufficient statistical power.The exact n for each experiment was described in corresponding figure legends.
Data exclusions	No data was excluded from the analyses.
Replication	Most data were representative of three independent experiments.
Randomization	Animals were randomly allocated to experimental groups. All animal experiments use mice with matched age.
Blinding	Not applicable, as no human research in this manuscript.

Reporting for specific materials, systems and methods

We require information from authors about some types of materials, experimental systems and methods used in many studies. Here, indicate whether each material, system or method listed is relevant to your study. If you are not sure if a list item applies to your research, read the appropriate section before selecting a response.

Materials & experimental systems

n/a	Involved in the study
	☒ Antibodies
	☒ Eukaryotic cell lines
☒	Palaeontology and archaeology
	☒ Animals and other organisms
☒	Clinical data
☒	Dual use research of concern
☒	Plants

Methods

n/a	Involved in the study
☒	ChIP-seq
	☒ Flow cytometry
☒	MRI-based neuroimaging

Antibodies

Antibodies used

GAPHD (Proteintech, 60004-1-Ig, 1:5000), TFR1 (Proteintech, 66180-1-Ig, 1:2000), FTH1 (HUABIO, ET1705-55, 1:1000), FTL (HUABIO, EM1707-17, 1:1000), DMT1 (HUABIO, ER1907-55, 1:1000), FPN1 (HUABIO, HA601178, 1:1000), PKCβ (Proteintech, 12919-1-AP, 1:2000), PKCβI (Santa Cruz, sc-8049,1:500), PKCβII (Santa Cruz, sc-13149, 1:500), AAK1 (CST, 79832T, 1:2000), AP2M1 (Abclonal, A11070, 1:2000), Flag (Abcepta, AP1013A, 1:1000), p-Ser/Thr (Abcam, ab17464, 1:3000), p-AP2M1(Thr156) (Abcam, ab109397, 1:3000).

Validation

All antibodies used in this study are commercially available and all are validated by the vendors for the specific assays and species used; the validation data is available on the vendors website.
1.GAPHD (Proteintech, 60004-1-Ig, 1:5000)
https://www.ptgcn.com/products/GAPDH-Antibody-60004-1-Ig.htm
The manufacturer has validated this antibody for in vivo use in all.
2.TFR1 (Proteintech, 66180-1-Ig, 1:2000)
https://www.ptgcn.com/products/CD71-Antibody-66180-1-Ig.htm
The manufacturer has validated this antibody for in vivo use in species human.
3.FTH1 (HUABIO, ET1705-55, 1:1000)
https://huabio.cn/products/Ferritin-Heavy-Chain-antibody-ET1705-55
The manufacturer has validated this antibody for in vivo use in species human.
4.FTL (HUABIO, EM1707-17, 1:1000)
https://huabio.cn/products/FTL-antibody-EM1707-17
The manufacturer has validated this antibody for in vivo use in species human.
5.DMT1 (HUABIO, ER1907-55, 1:1000)
https://huabio.cn/products/DMT1-antibody-ER1907-55
The manufacturer has validated this antibody for in vivo use in all.
6.FPN1 (HUABIO, HA601178, 1:1000)
https://huabio.cn/products/SLC40A1-antibody-HA601178
The manufacturer has validated this antibody for in vivo use in all.
7.PKCβ (Proteintech, 12919-1-AP, 1:2000)
https://www.ptgcn.com/products/PRKCB-Antibody-12919-1-AP.htm
The manufacturer has validated this antibody for in vivo use in all.
8.PKCβI (Santa Cruz, sc-8049, 1:500)
https://www.scbt.com/zh/p/pkc-betai-antibody-e-3
The manufacturer has validated this antibody for in vivo use in all.
9.PKCβII (Santa Cruz, sc-13149, 1:500)
https://www.scbt.com/zh/p/pkc-betaii-antibody-f-7
The manufacturer has validated this antibody for in vivo use in all.
10.AAK1 (CST, 79832T, 1:2000)
https://www.cellsignal.cn/products/primary-antibodies/aak1-antibody/79832
The manufacturer has validated this antibody for in vivo use in all.
11.AP2M1 (Abclonal, A11070, 1:2000)
https://abclonal.com.cn/catalog/A11070
The manufacturer has validated this antibody for in vivo use in all.
12.Flag (Abcepta, AP1013A, 1:1000)
https://www.abcepta.com.cn/products/AP1013a-DYKDDDDK-FLAG-Tag-Antibody
The manufacturer has validated this antibody for in vivo use in species human.
13.p-Ser/Thr (Abcam, ab17464, 1:3000)
https://www.abcam.cn/products/primary-antibodies/phospho-ser-thr-phe-antibody-epr26858-4-ab300625
The manufacturer has validated this antibody for in vivo use in species human.
14.p-AP2M1(Thr156) (Abcam, ab109397, 1:3000)
https://www.abcam.cn/products/primary-antibodies/ap2m1-phospho-t156-antibody-epr4700-ab109397
The manufacturer has validated this antibody for in vivo use in species human.

Eukaryotic cell lines

Policy information about cell lines and Sex and Gender in Research

Cell line source(s)	MDA-MB-231 (Cat# HTB-26, ATCC), HT1080 (Cat# CCL-121, ATCC), MCF7 (Cat# HTB-22, ATCC), CAL51 (Cat# ACC 302, DSMZ), A549 (Cat# CCL-185, ATCC), HN6 (Cat# CVCL-8129, Cellosaurus)
Authentication	All cell lines were authenticated.
Mycoplasma contamination	All cell lines were tested negative for mycoplasma contamination.
Commonly misidentified lines (See ICLAC register)	No commonly misidentified cell lines were used in the study.

Animals and other research organisms

Policy information about studies involving animals; ARRIVE guidelines recommended for reporting animal research, and Sex and Gender in Research

Laboratory animals	Female-5-week-old Balb/c nude mice were obtained from Guangdong Medical Laboratory Animal Center.<br>All mice were monitored for tumor formation, with tumor length and width measured every four days using calipers. Tumor volume and body weight were recorded. Volumes were calculated using the formula: $0.5 \times a \times b^2$ (in millimeters), where a represents the length and b the width. Mice were euthanized at the following humane end points: a total tumor volume exceeding 2 cm3, tumor ulceration, necrotic tissue or a weight loss greater than 10% of the initial weight. Euthanasia was performed via cervical dislocation following isoflurane anesthesia. After mice were sacrificed, the tumor tissues were excised and weighed. All procedures involving mice and experimental protocols were approved by Institutional Animal Care and Use Committee (IACUC) of Sun Yat-sen University Cancer Center.
Wild animals	No wild animals were used in the study.
Reporting on sex	Sex and gender were not considered in study design.
Field-collected samples	No field-collected samples were used in the study.
Ethics oversight	All procedures involving mice and experimental protocols were approved by the Institutional Animal Care and Use Committee (IACUC) of Sun Yat-sen University Cancer Center.

Note that full information on the approval of the study protocol must also be provided in the manuscript.

Plants

Seed stocks	N/A
Novel plant genotypes	N/A
Authentication	N/A

Flow Cytometry

Plots

Confirm that:

- [x] The axis labels state the marker and fluorochrome used (e.g. CD4-FITC).
- [x] The axis scales are clearly visible. Include numbers along axes only for bottom left plot of group (a 'group' is an analysis of identical markers).
- [x] All plots are contour plots with outliers or pseudocolor plots.
- [x] A numerical value for number of cells or percentage (with statistics) is provided.

Methodology

Sample preparation	For immunoblots, cells were collected and washed with 4°C PBS, followed by lysing with RIPA buffer containing protease inhibitors and phosphatase inhibitors.<br>For immunofluorescent staining, cells which have been planted in a 35mm plate were washed and stained with indicated

	specific-antibody for 60 min on ice, followed by incubation of fluorescent antibodies for 30min and washed with PBS.<br>For iron level detection, cells which have been planted in a 10cm plate were collected and treated with lysis buffer for 15min on ice. The indicated buffer was contained in the iron assay kits (Elabscience, E-BC-K880-M-96T; Elabscience, E-BC-K881-M-96T)<br>For lipid peroxidation assays, cells which have been planted in a 6-well plate were washed with 4°C PBS and treated with 5μM BODIPY 581/591 C11 (Invitrogen, D3861) for 30 min in 37°C.<br>For cell death assays, cells which have been planted in a 6-well plate were washed with 4°C PBS and treated with 5μg/ml propidium iodide for 5 min in 37°C.<br>For endocytosis assays, cells were collected and washed with RPMI 1640 containing 7%FBS and 30mM HEPES, followed by incubation with TFR1-specific antibody for 60min on ice.
Instrument	All flow cytometry analyses were conducted on CytoFLEX (Beckman).
Software	The data were analysed using FlowJo software (FlowJo Vx.10.0.7), Kaluza Analysis software (Kaluza Analysis Version 2.1), or CytExpert software (CytExpert 2.4) according to manufacturers' instructions.
Cell population abundance	No cell sorting was performed in the study.
Gating strategy	For all experiments, cells were first gated by FSC/SSC to exclude debris. Then, target cell population for further analysis were gated by cell surface marker (e.g. human CD3). For surface marker and intracellular cytokine staining, isotype control antibodies were used to define background and non-specific binding signal.

☒ Tick this box to confirm that a figure exemplifying the gating strategy is provided in the Supplementary Information.
