# Supplementary material for: AAK1 activation-mediated iron trafficking drives ferroptotic cell death
Source: Nat Commun. 2025 Dec 17;17:819. doi: 10.1038/s41467-025-67523-9 (PMC12824188; doi:10.1038/s41467-025-67523-9)

Fig.1A

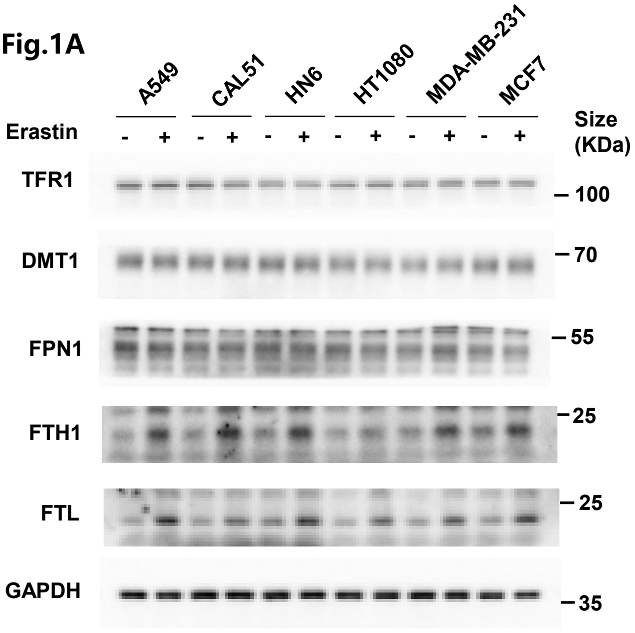

Fig.1F

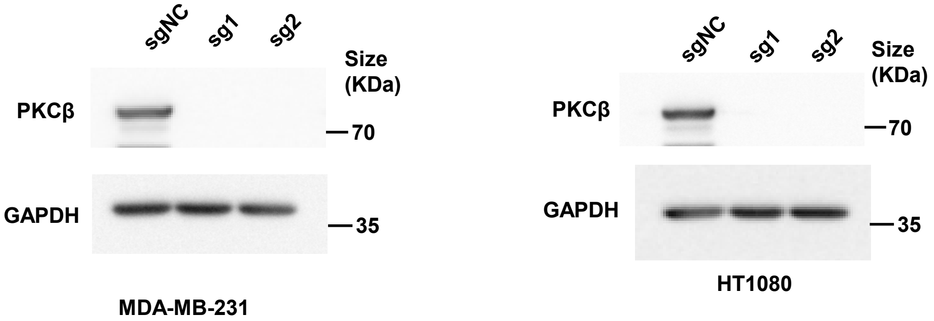

Fig.1J

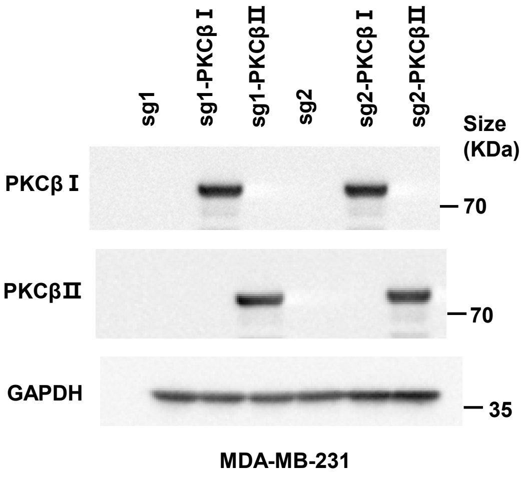

Fig.1K

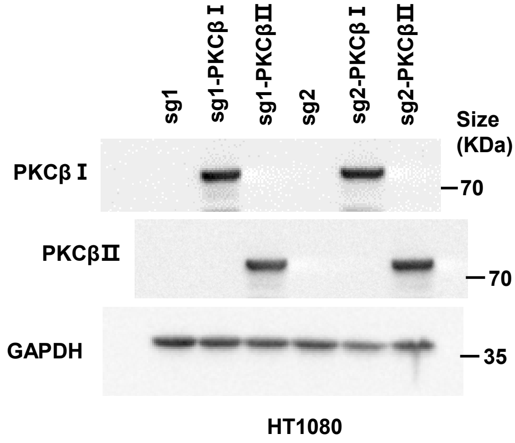

Fig.2B

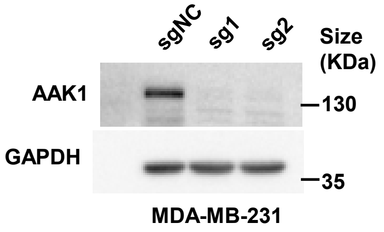

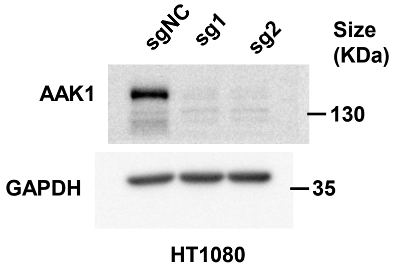

Fig.2E

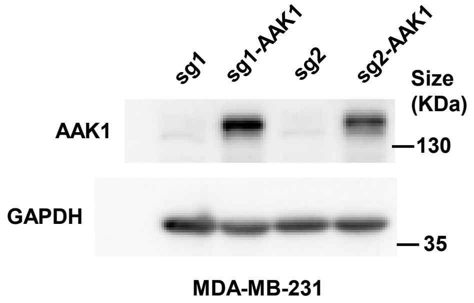

Fig.2F

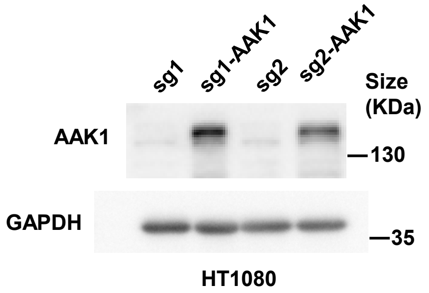

Fig.2H

Rep.1

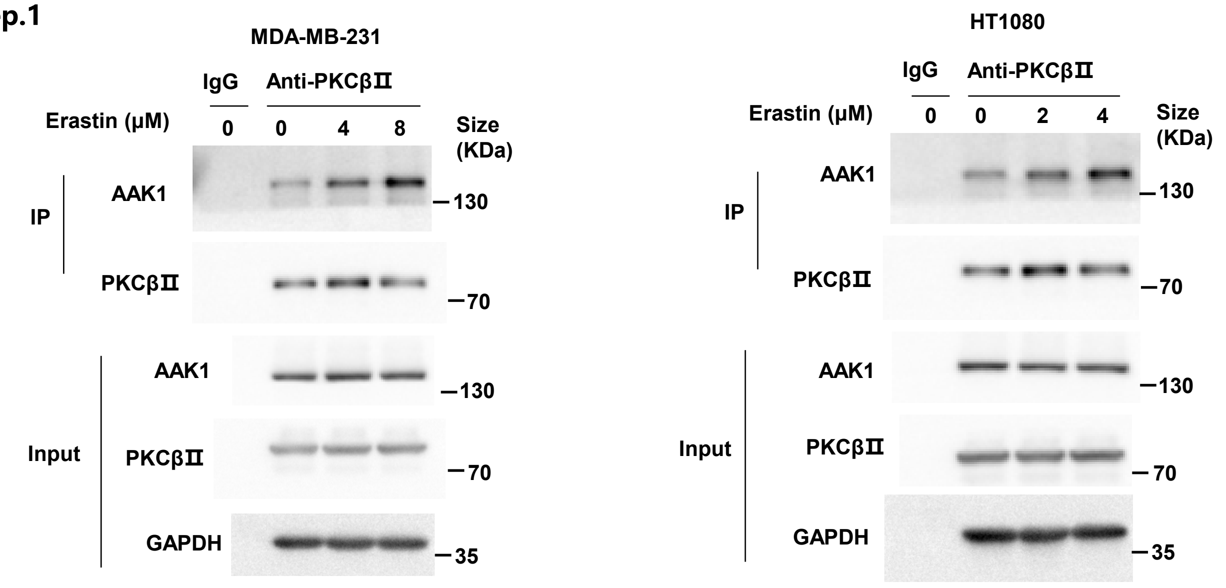

Rep.2

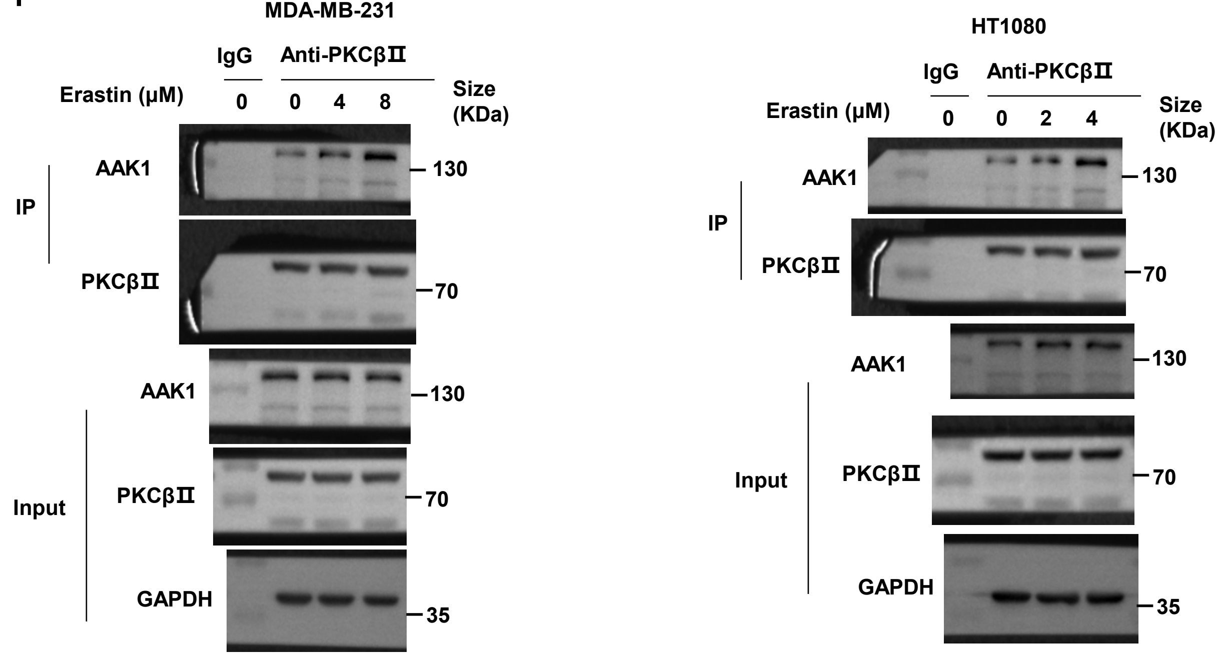

Rep.3

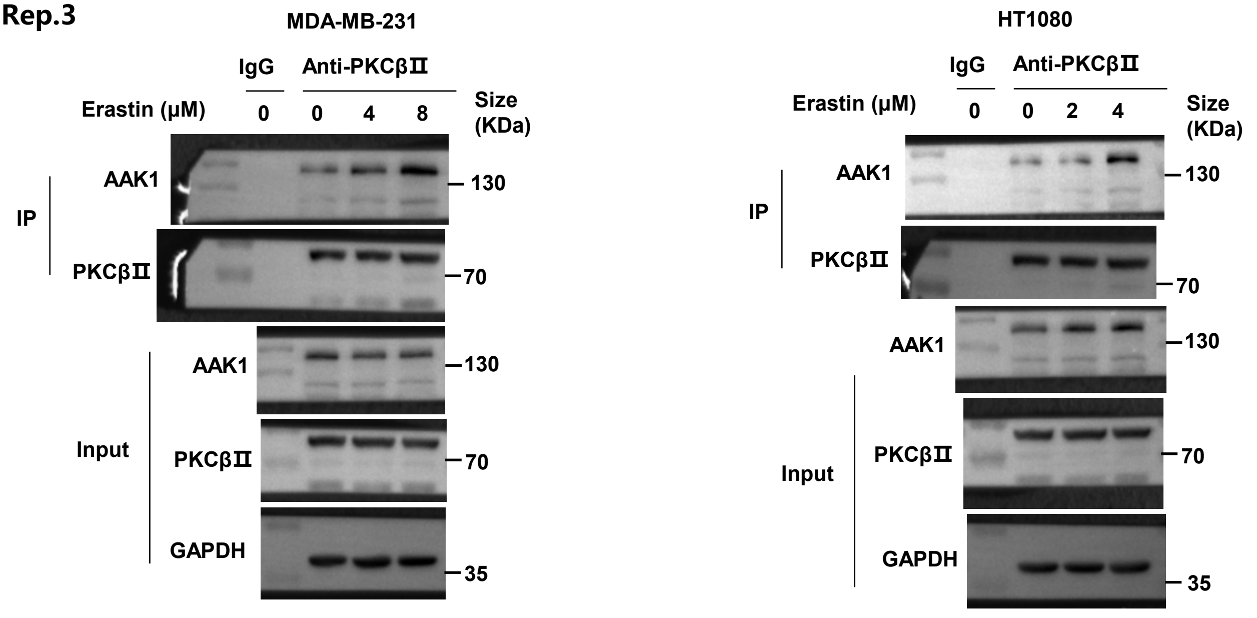

Fig.2I
Rep.1

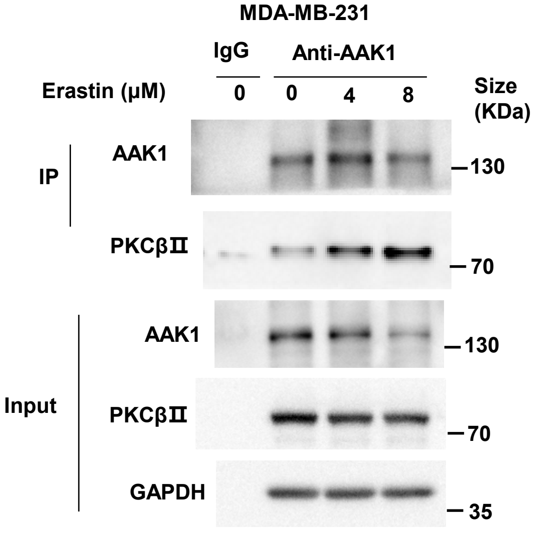

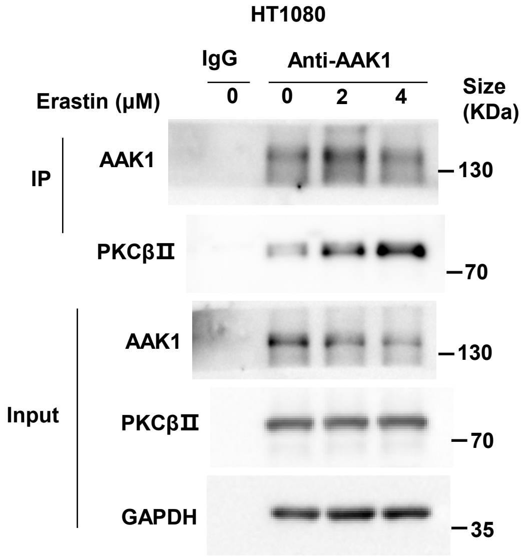

Rep.2

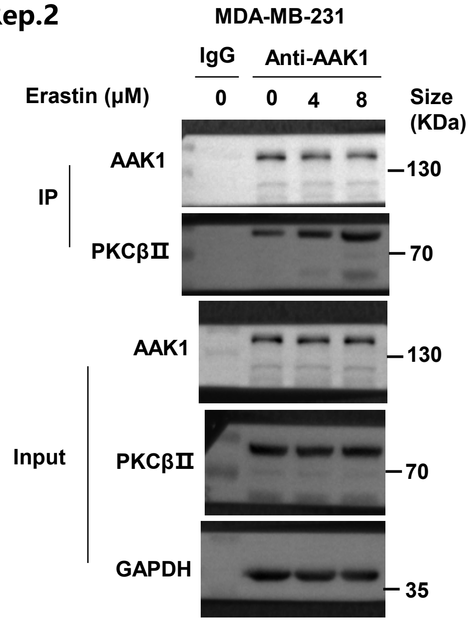

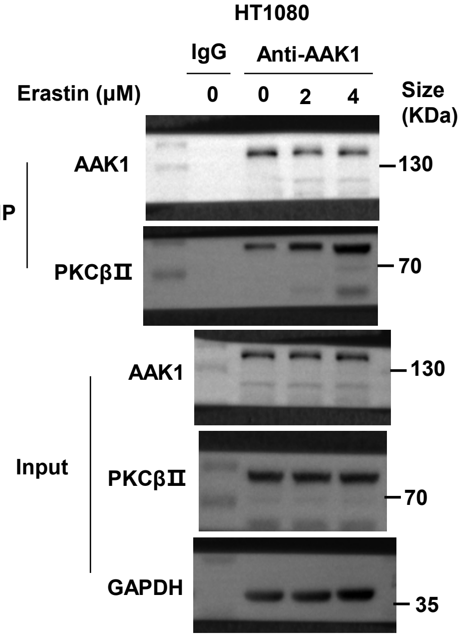

Rep.3

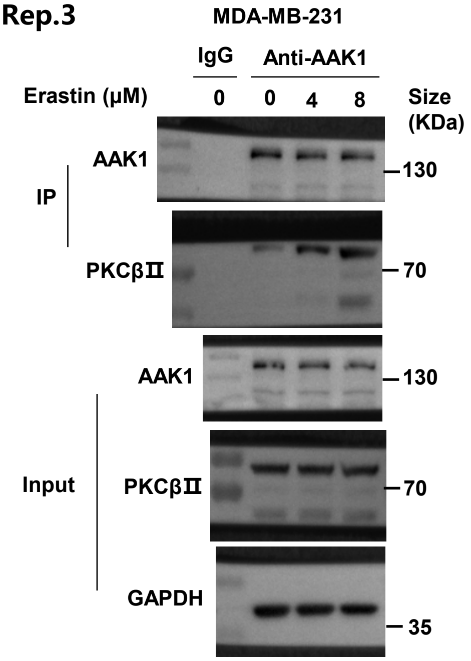

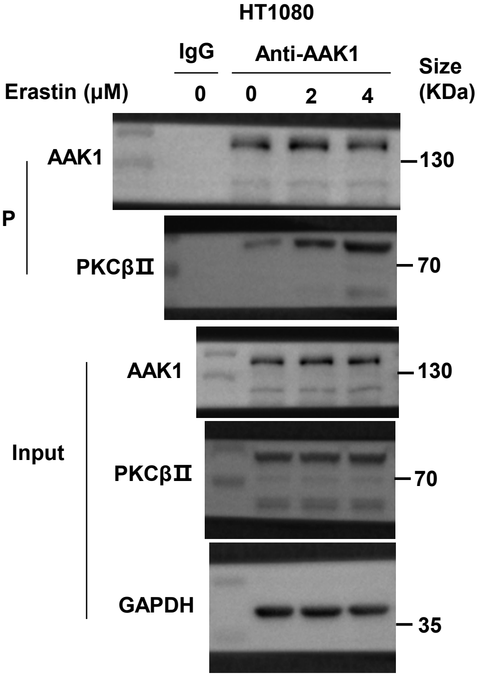

Fig. 3A

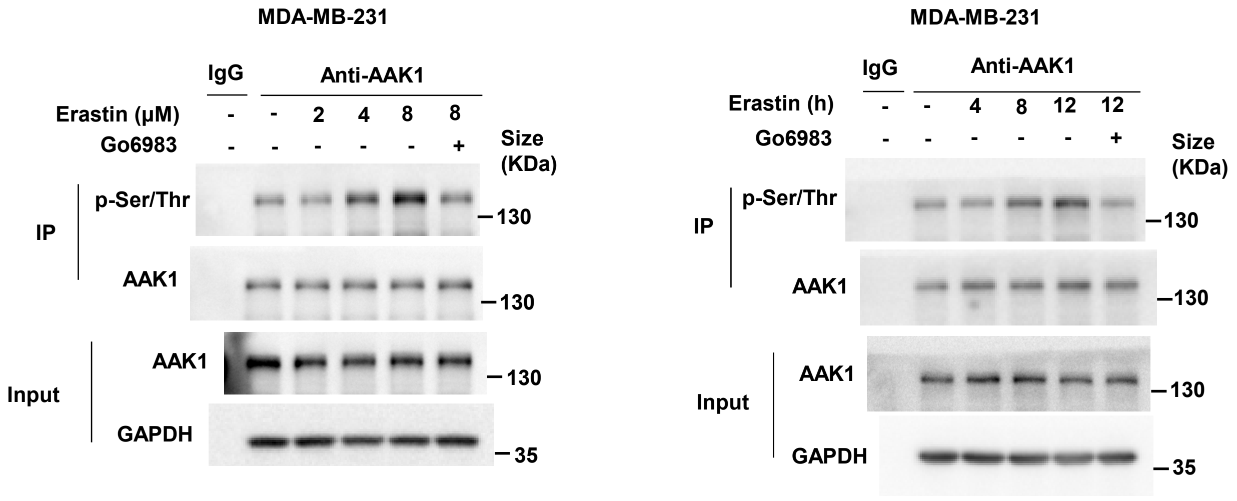
MDA-MB-231
IgG
Anti-AAK1
Erastin (μM) - - 2 4 8 8
Go6983 - - - - - +
Size (KDa)
IP
p-Ser/Thr
AAK1
Input
AAK1
GAPDH
130
130
130
35
MDA-MB-231
IgG
Anti-AAK1
Erastin (h) - - 4 8 12 12
Go6983 - - - - - +
Size (KDa)
IP
p-Ser/Thr
AAK1
Input
AAK1
GAPDH
130
130
130
35

Fig. 3B

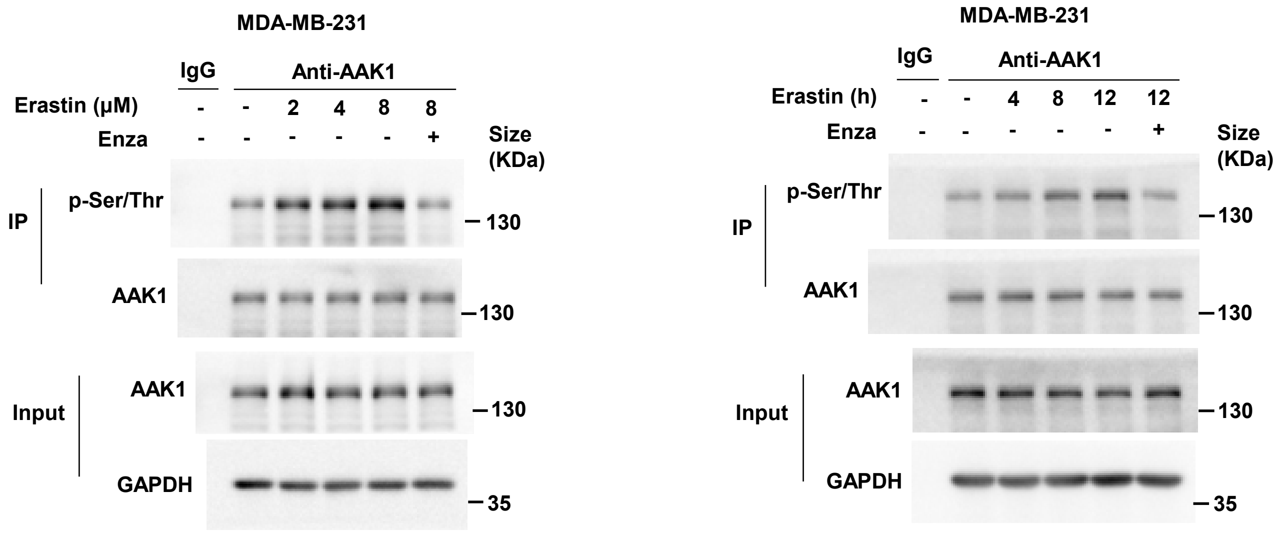
MDA-MB-231
IgG
Anti-AAK1
Erastin (μM) - - 2 4 8 8
Enza - - - - - +
Size (KDa)
IP
p-Ser/Thr
AAK1
Input
AAK1
GAPDH
130
130
130
35
MDA-MB-231
IgG
Anti-AAK1
Erastin (h) - - 4 8 12 12
Enza - - - - - +
Size (KDa)
IP
p-Ser/Thr
AAK1
Input
AAK1
GAPDH
130
130
130
35

Fig. 3C

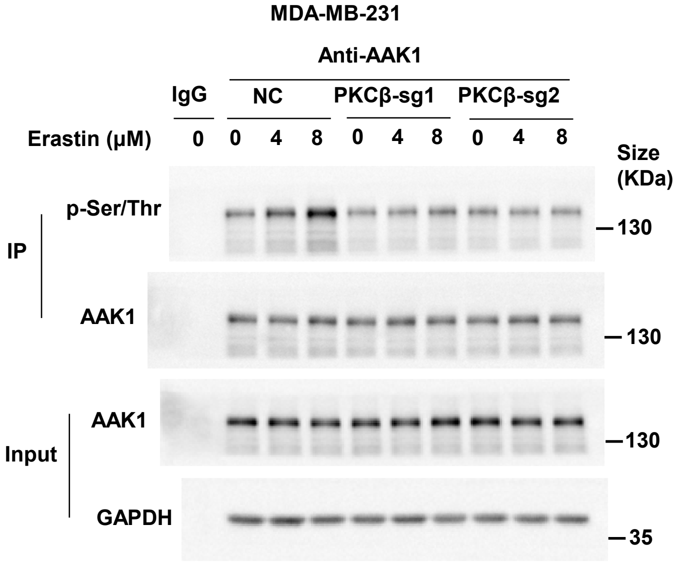
MDA-MB-231
Anti-AAK1
IgG
NC
PKCβ-sg1
PKCβ-sg2
Erastin (μM)
0 0 4 8 0 4 8 0 4 8
Size (KDa)
p-Ser/Thr
IP
130
AAK1
130
AAK1
Input
130
GAPDH
35

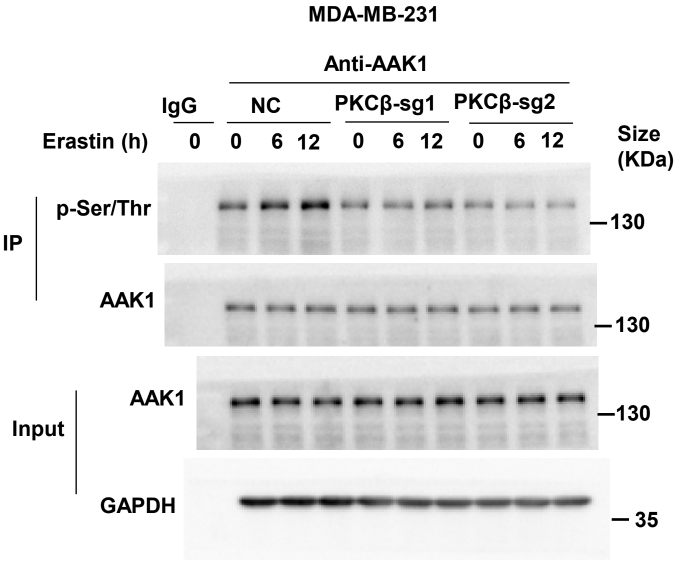
MDA-MB-231
Anti-AAK1
IgG
NC
PKCβ-sg1
PKCβ-sg2
Erastin (h)
0 0 6 12 0 6 12 0 6 12
Size (KDa)
p-Ser/Thr
IP
130
AAK1
130
AAK1
Input
130
GAPDH
35

Fig. 3D

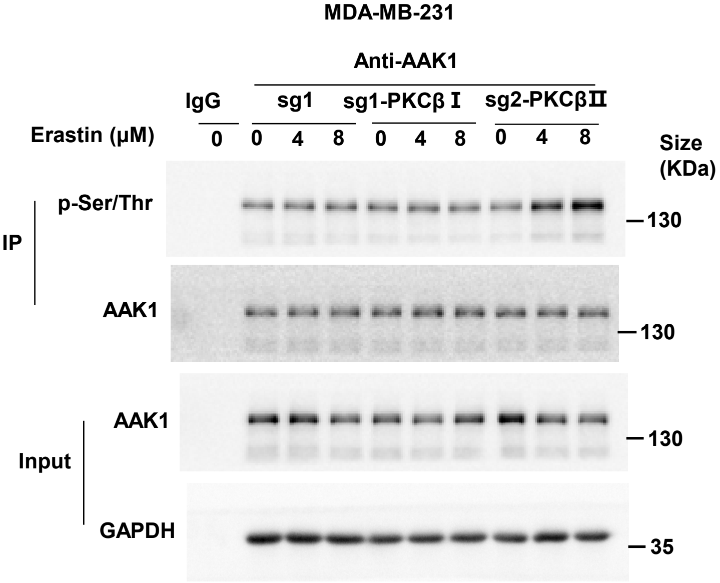
MDA-MB-231
Anti-AAK1
IgG
sg1
sg1-PKCβ I
sg2-PKCβII
Erastin (μM)
0
0 4 8
0 4 8
0 4 8
Size (KDa)
IP
p-Ser/Thr
AAK1
Input
AAK1
GAPDH
130
130
130
35

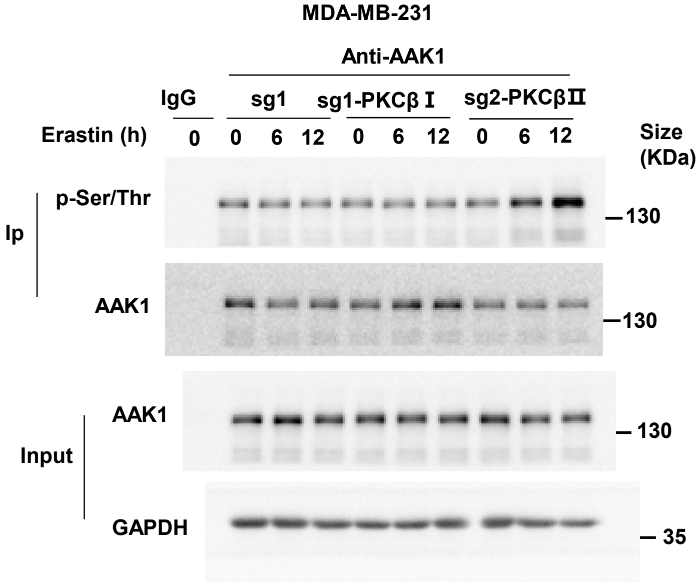
MDA-MB-231
Anti-AAK1
IgG
sg1
sg1-PKCβ I
sg2-PKCβII
Erastin (h)
0
0 6 12
0 6 12
0 6 12
Size (KDa)
Ip
p-Ser/Thr
AAK1
Input
AAK1
GAPDH
130
130
130
35

Fig. 3E

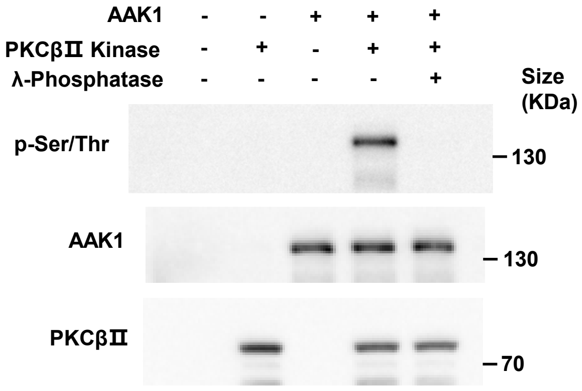

Fig. 3F

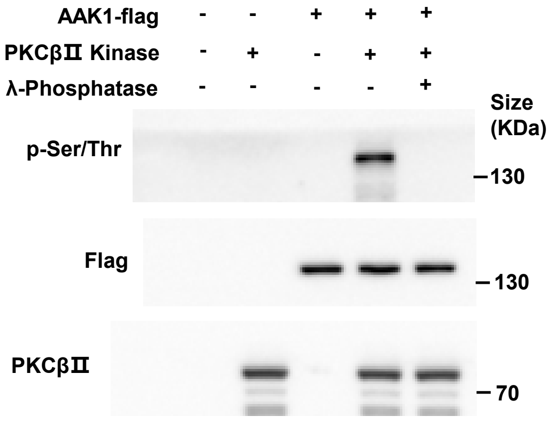

Fig. 3H

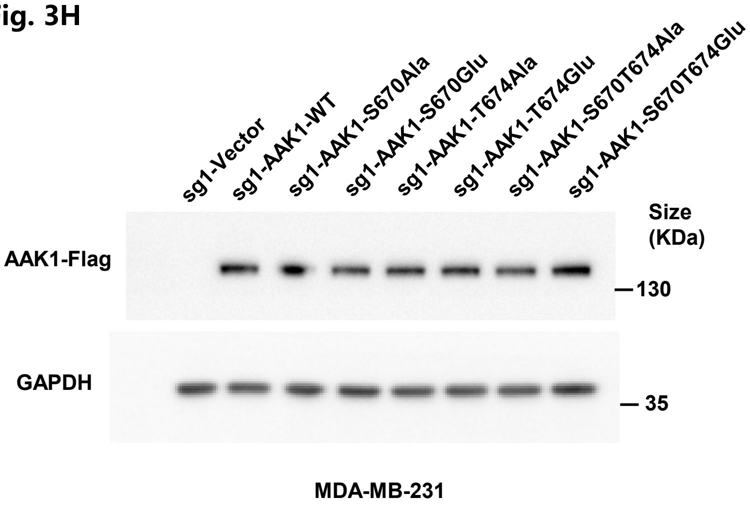

Fig. 3I

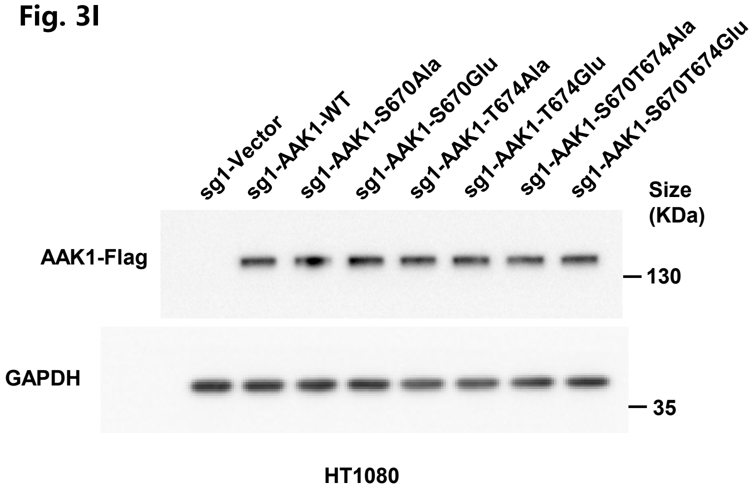

Fig. 3K

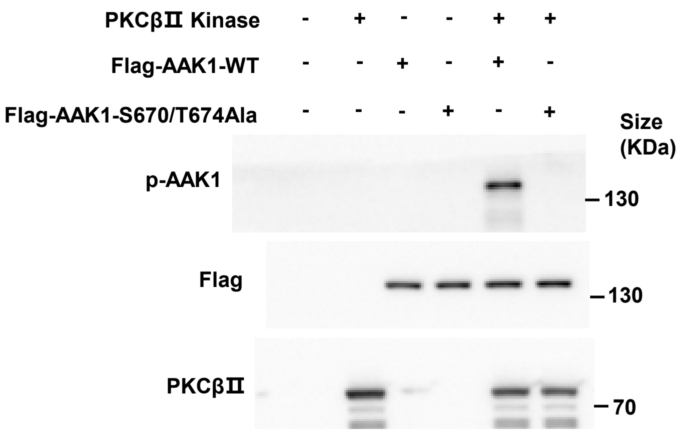

Fig. 3L

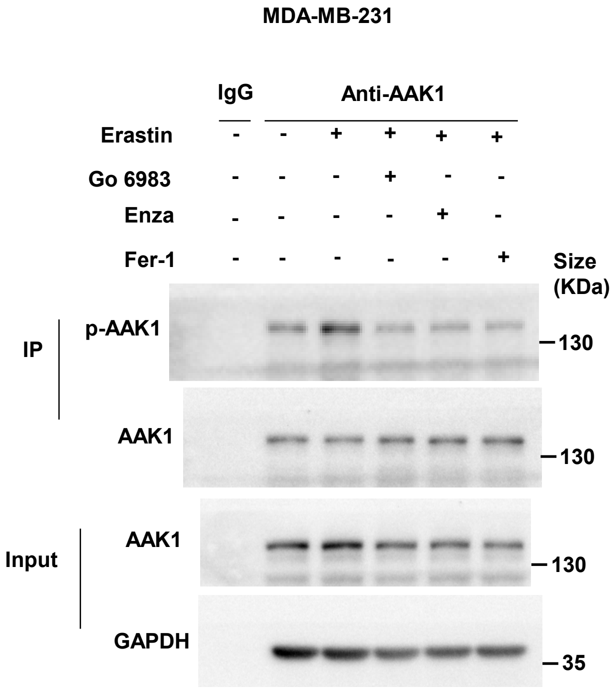

Fig. 3M

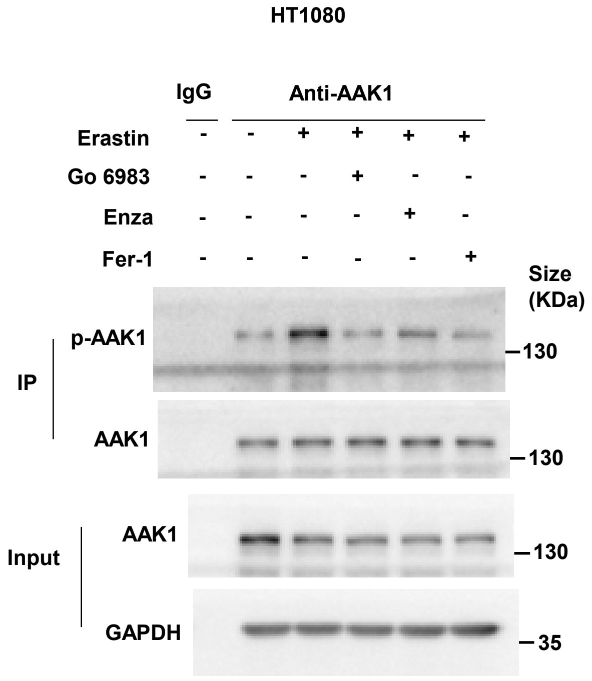

Fig. 4A

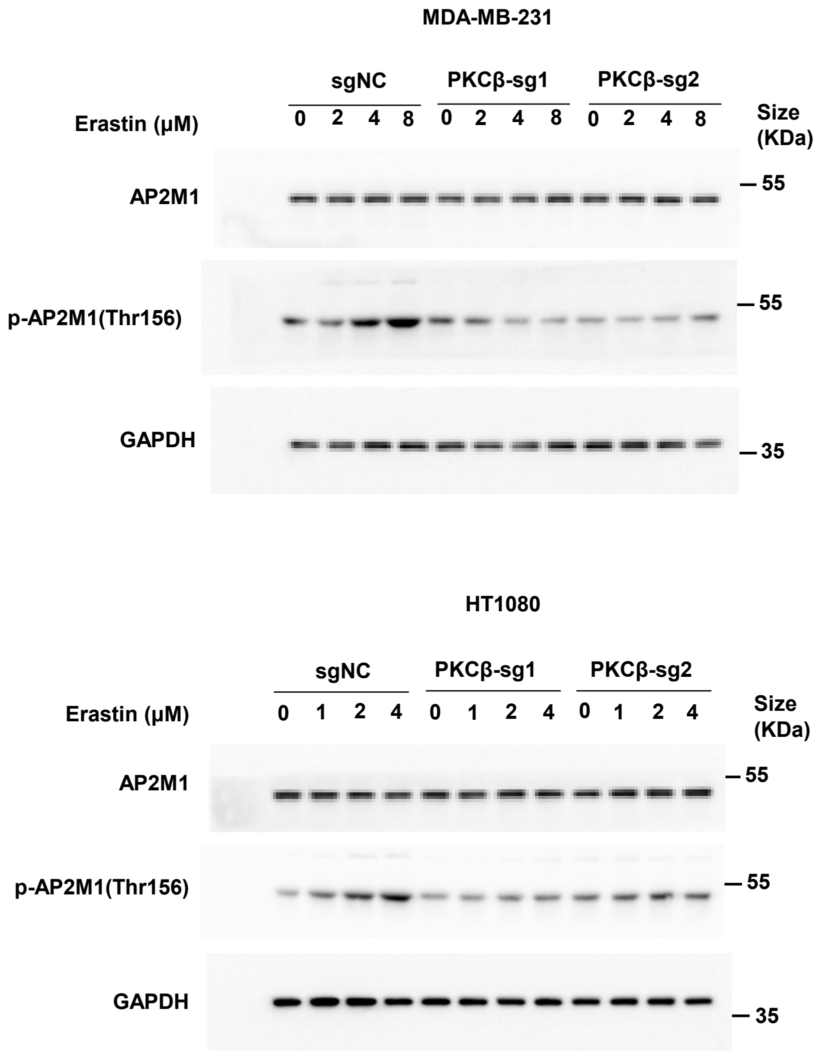
MDA-MB-231
sgNC
PKCβ-sg1
PKCβ-sg2
Erastin (μM)
0 2 4 8 0 2 4 8 0 2 4 8
Size (KDa)
AP2M1
p-AP2M1(Thr156)
GAPDH
55
55
35
HT1080
sgNC
PKCβ-sg1
PKCβ-sg2
Erastin (μM)
0 1 2 4 0 1 2 4 0 1 2 4
Size (KDa)
AP2M1
p-AP2M1(Thr156)
GAPDH
55
55
35

Fig. 4B

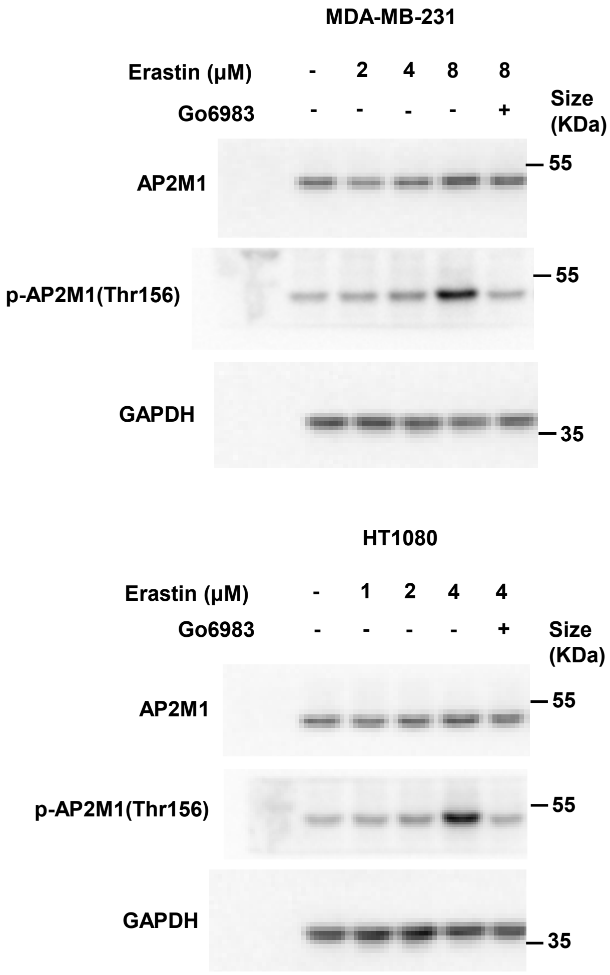
MDA-MB-231
Erastin (μM) - 2 4 8 8
Go6983 - - - - +
Size (KDa)
AP2M1
55
p-AP2M1(Thr156)
55
GAPDH
35
HT1080
Erastin (μM) - 1 2 4 4
Go6983 - - - - +
Size (KDa)
AP2M1
55
p-AP2M1(Thr156)
55
GAPDH
35

Fig. 4C

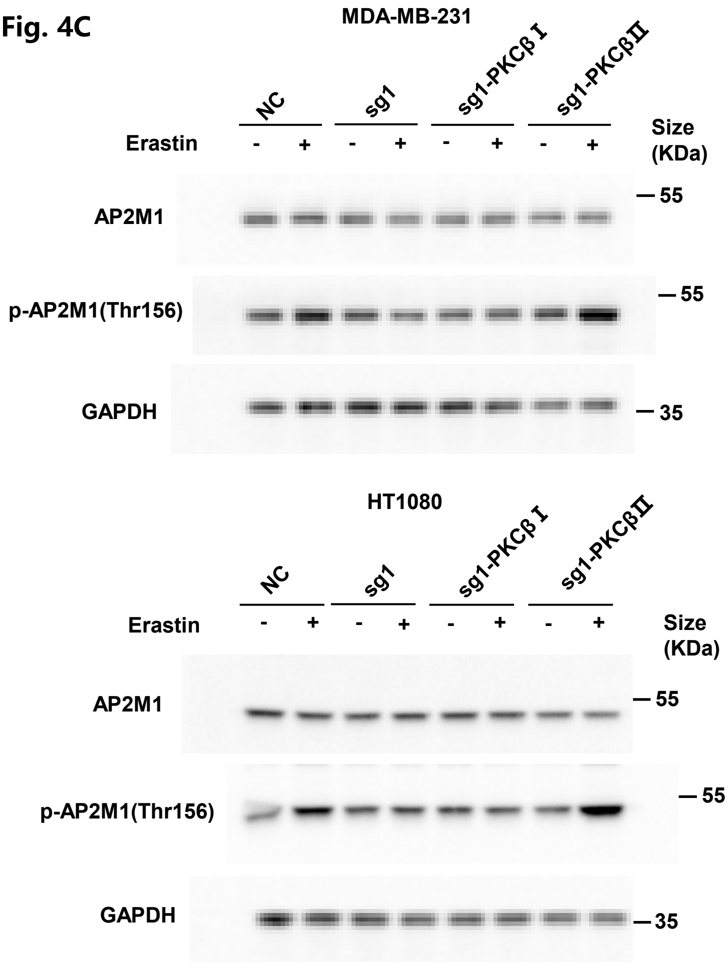
MDA-MB-231
NC
sg1
sg1-PKCβ I
sg1-PKCβII
Erastin - + - + - + - +
Size (KDa)
AP2M1
55
p-AP2M1(Thr156)
55
GAPDH
35
HT1080
NC
sg1
sg1-PKCβ I
sg1-PKCβII
Erastin - + - + - + - +
Size (KDa)
AP2M1
55
p-AP2M1(Thr156)
55
GAPDH
35

Fig. 4D

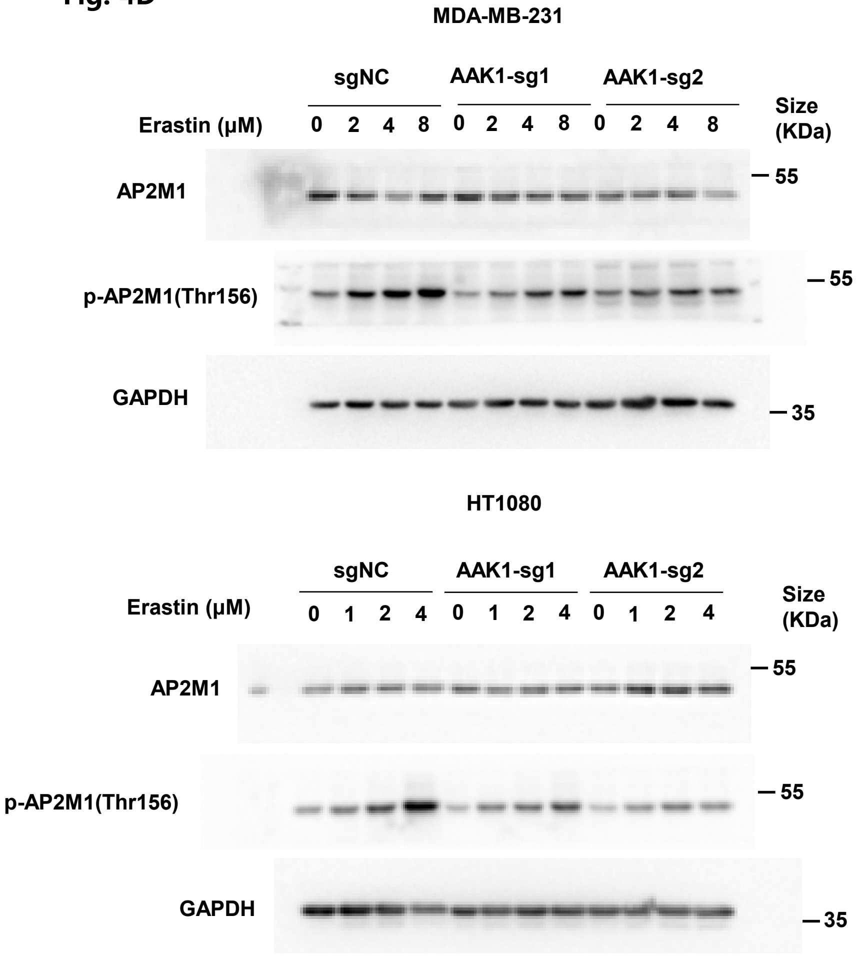
MDA-MB-231
sgNC
AAK1-sg1
AAK1-sg2
Erastin (μM)
0 2 4 8 0 2 4 8 0 2 4 8
Size (KDa)
AP2M1
55
p-AP2M1(Thr156)
55
GAPDH
35
HT1080
sgNC
AAK1-sg1
AAK1-sg2
Erastin (μM)
0 1 2 4 0 1 2 4 0 1 2 4
Size (KDa)
AP2M1
55
p-AP2M1(Thr156)
55
GAPDH
35

Fig. 4E

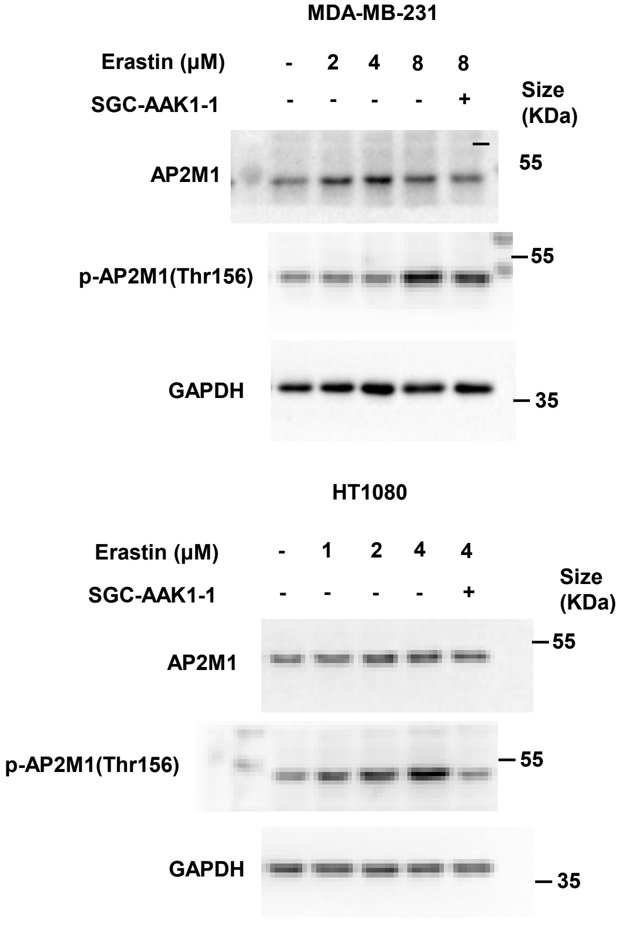
MDA-MB-231
Erastin (μM)
- 2 4 8 8
SGC-AAK1-1
- - - - +
Size (KDa)
AP2M1
55
p-AP2M1(Thr156)
55
GAPDH
35
HT1080
Erastin (μM)
- 1 2 4 4
SGC-AAK1-1
- - - - +
Size (KDa)
AP2M1
55
p-AP2M1(Thr156)
55
GAPDH
35

Fig. 4F

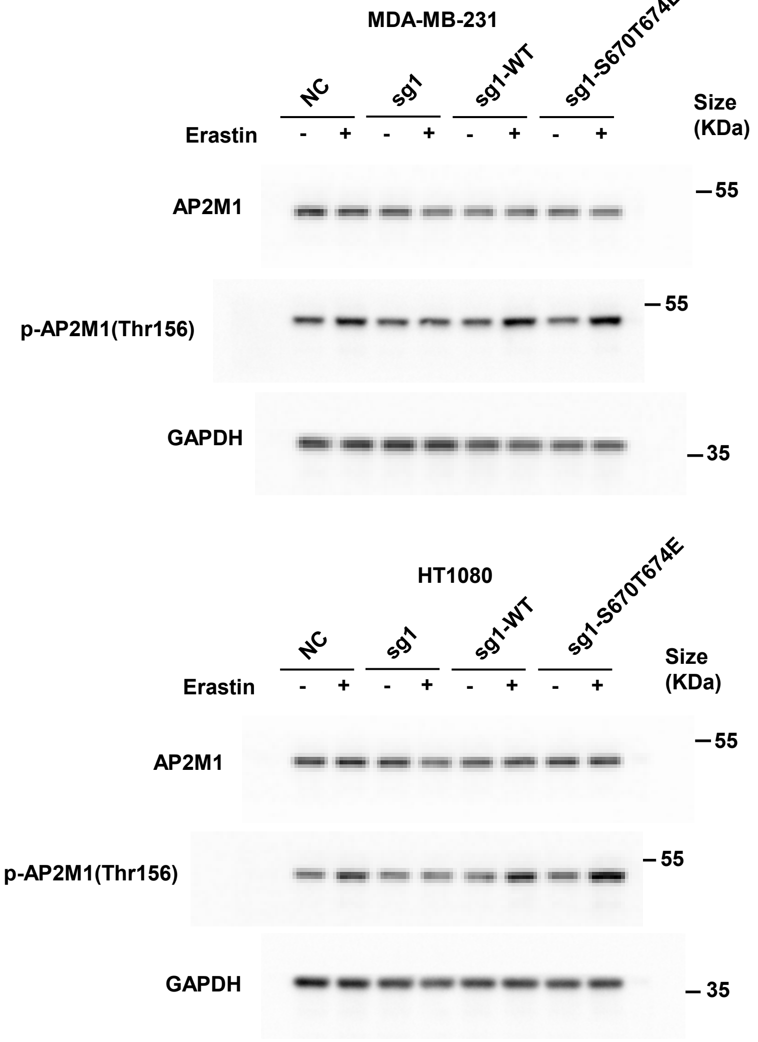
MDA-MB-231
NC
sg1
sg1-WT
sg1-S670T674E
Erastin
- + - + - + - +
Size
(KDa)
AP2M1
55
p-AP2M1(Thr156)
55
GAPDH
35
HT1080
NC
sg1
sg1-WT
sg1-S670T674E
Erastin
- + - + - + - +
Size
(KDa)
AP2M1
55
p-AP2M1(Thr156)
55
GAPDH
35

Fig. 4G

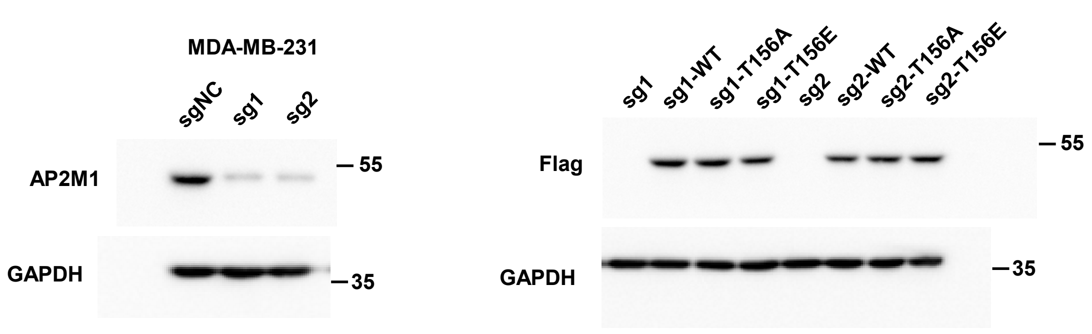
MDA-MB-231
sgNC
sg1
sg2
AP2M1
55
GAPDH
35
MDA-MB-231
sg1
sg1-WT
sg1-T156A
sg1-T156E
sg2
sg2-WT
sg2-T156A
sg2-T156E
Flag
55
GAPDH
35

Fig. 5A

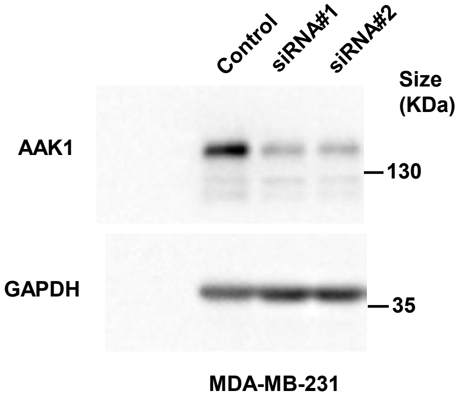
Control
siRNA#1
siRNA#2
Size (KDa)
AAK1
130
GAPDH
35
MDA-MB-231

Fig. 5B

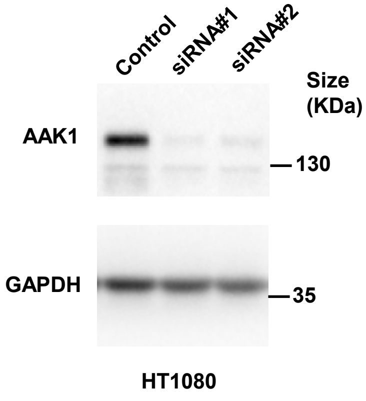
Control
siRNA#1
siRNA#2
Size (KDa)
AAK1
130
GAPDH
35
HT1080

Supplementary Fig. 4A

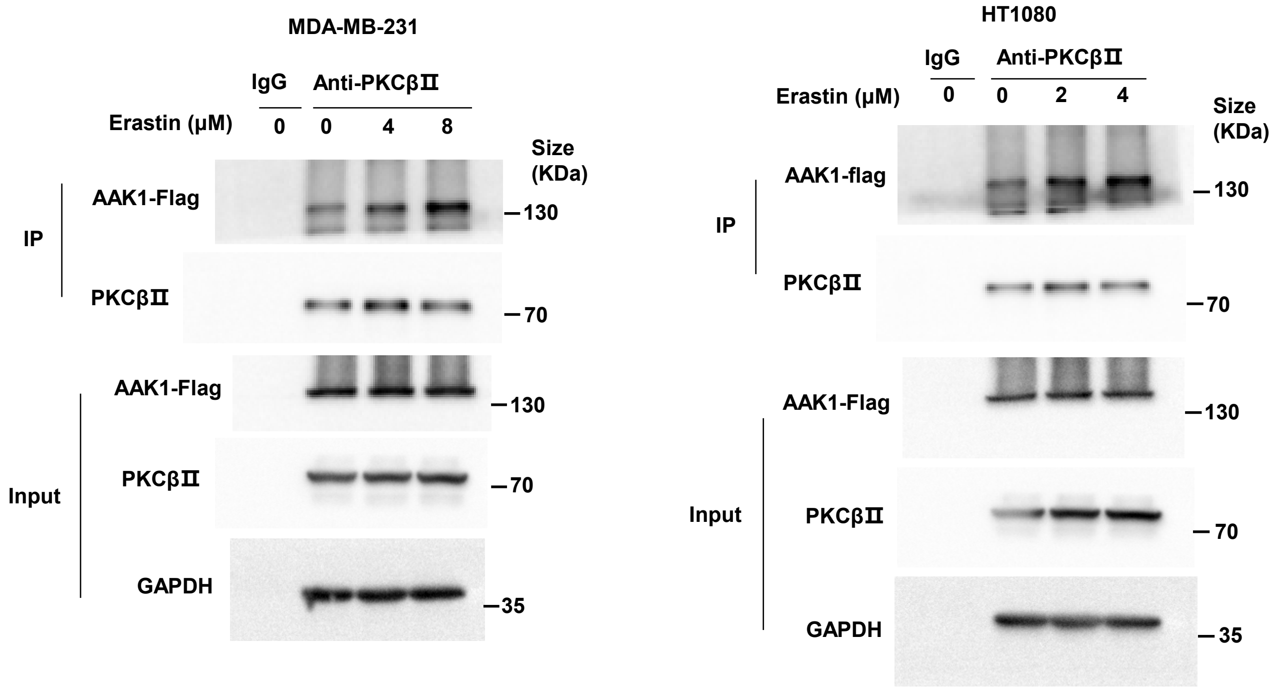

Supplementary Fig. 4B

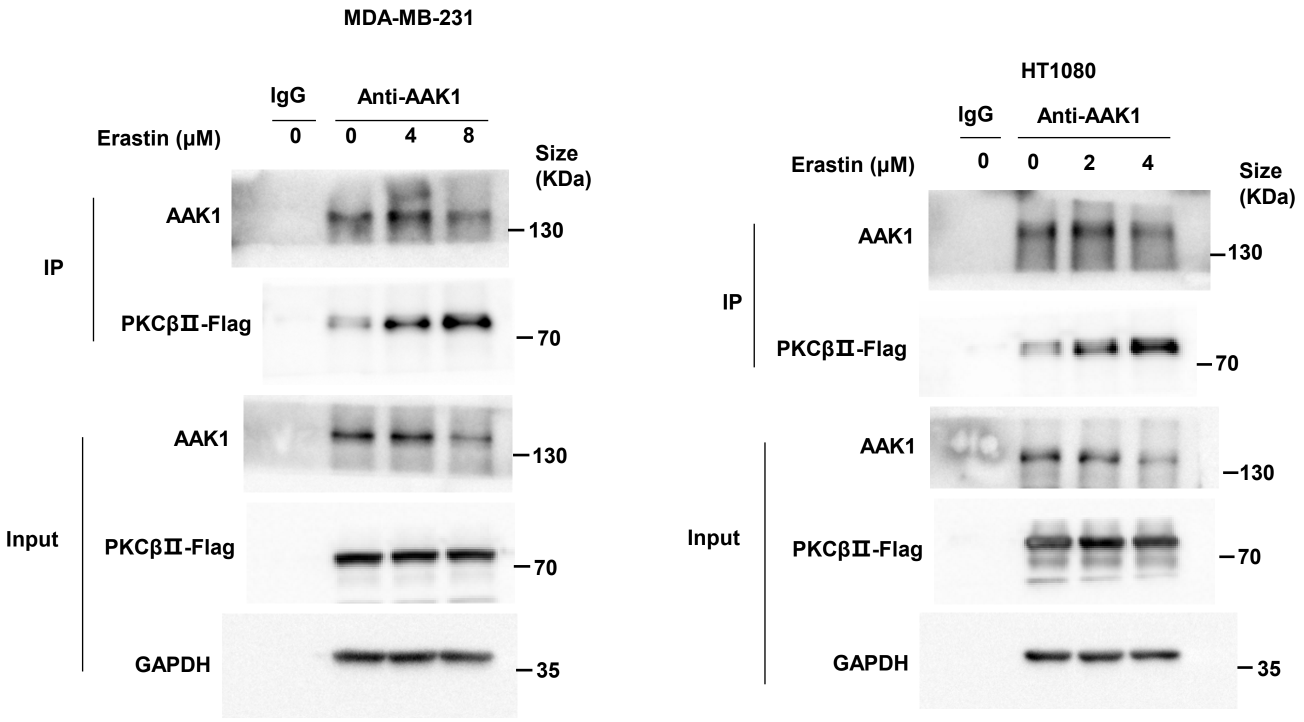

Supplementary Fig. 4C

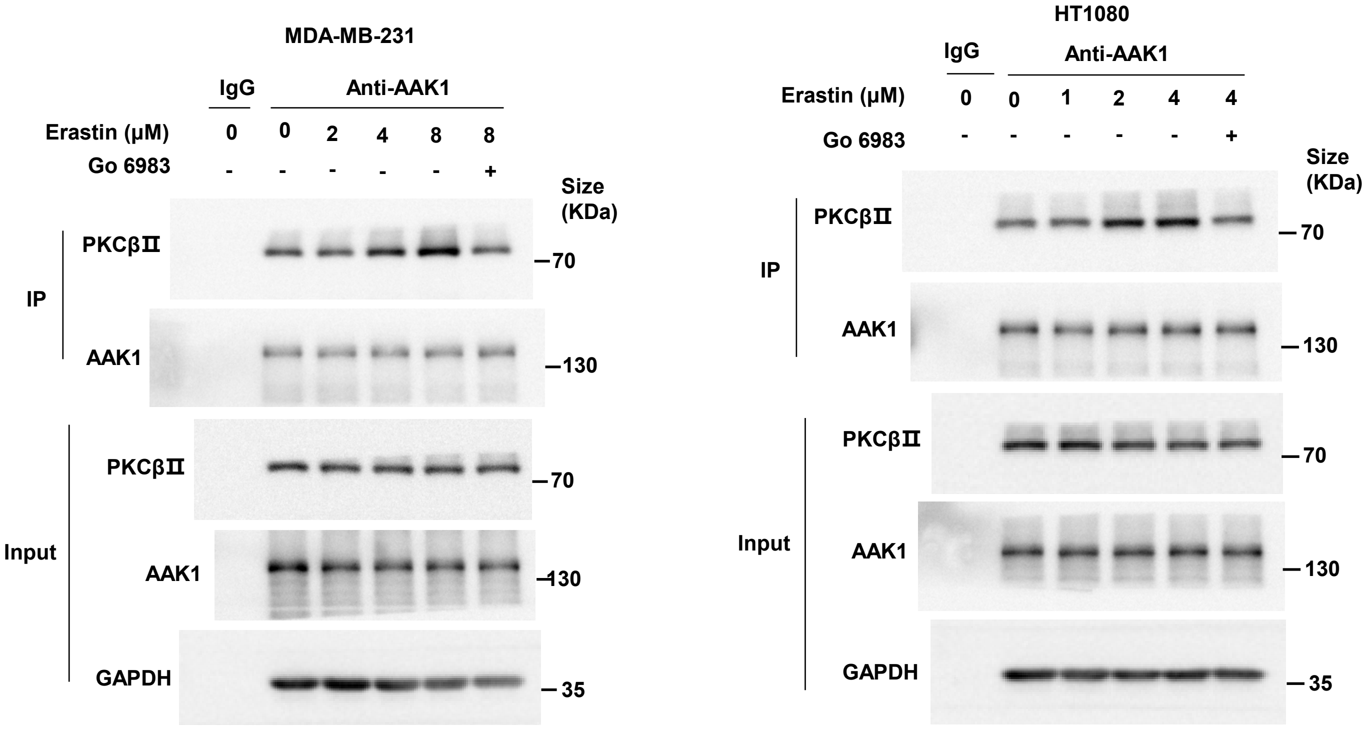

Supplementary Fig. 4D

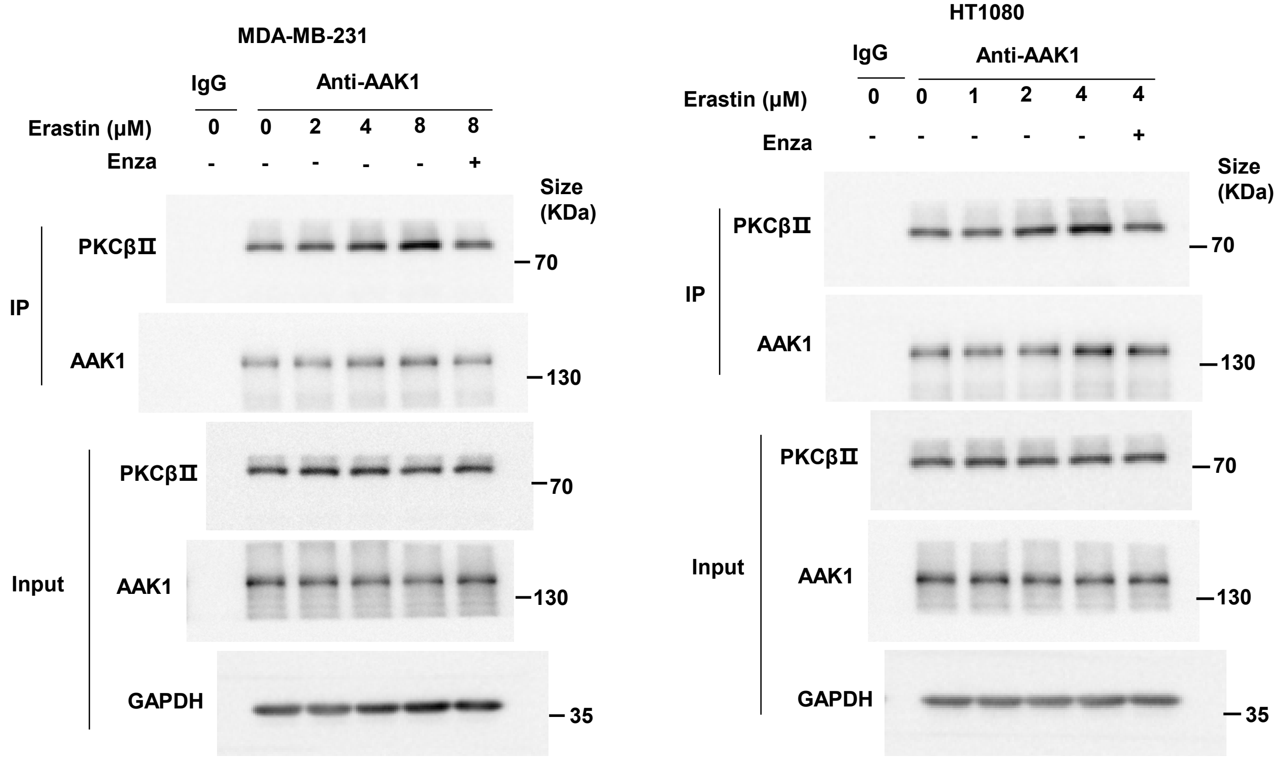

Supplementary Fig. 4E

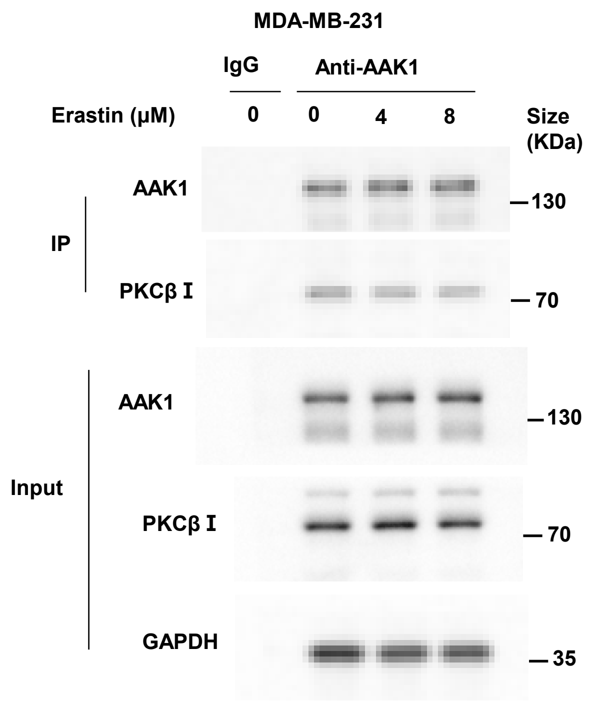

Supplementary Fig. 4F

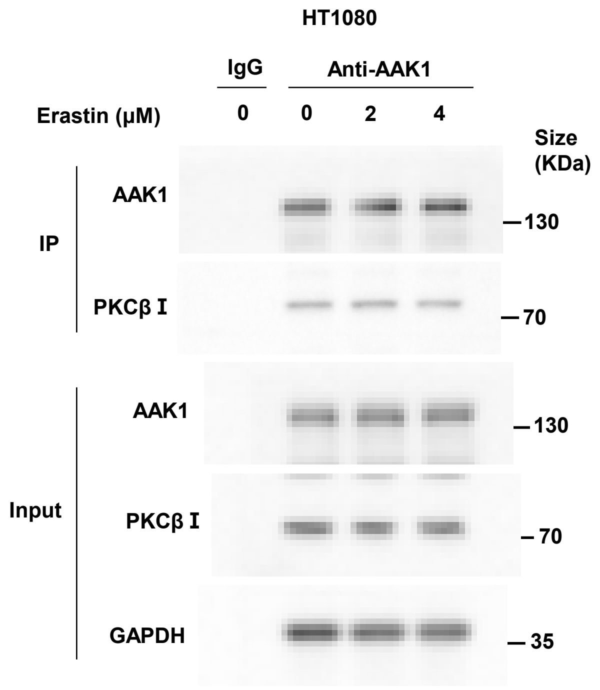

Supplementary Fig. 5A

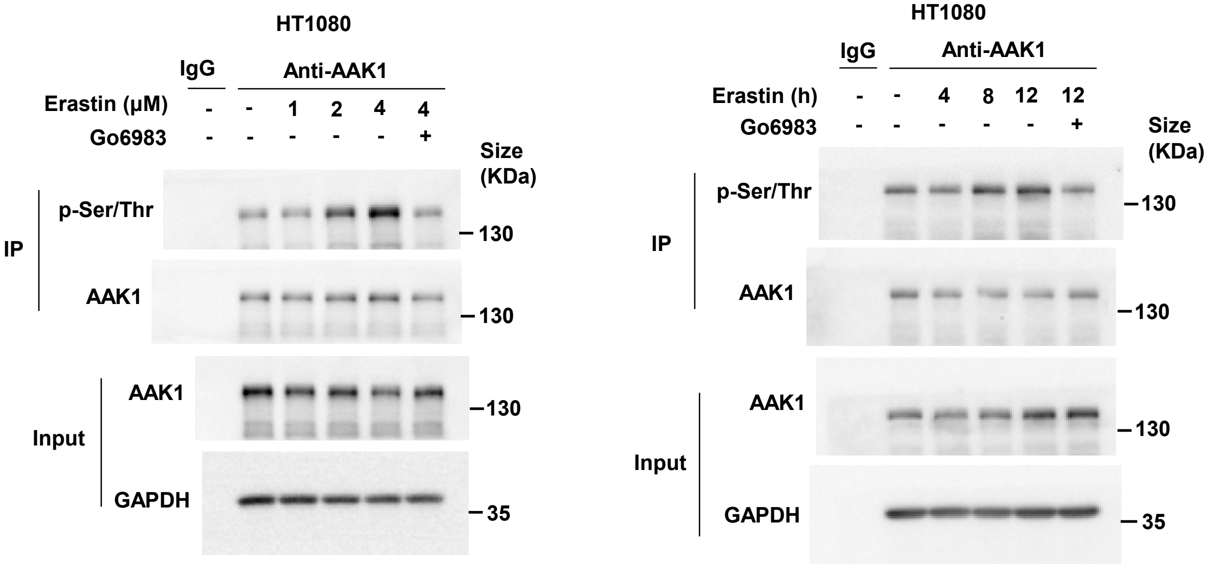

Supplementary Fig. 5B

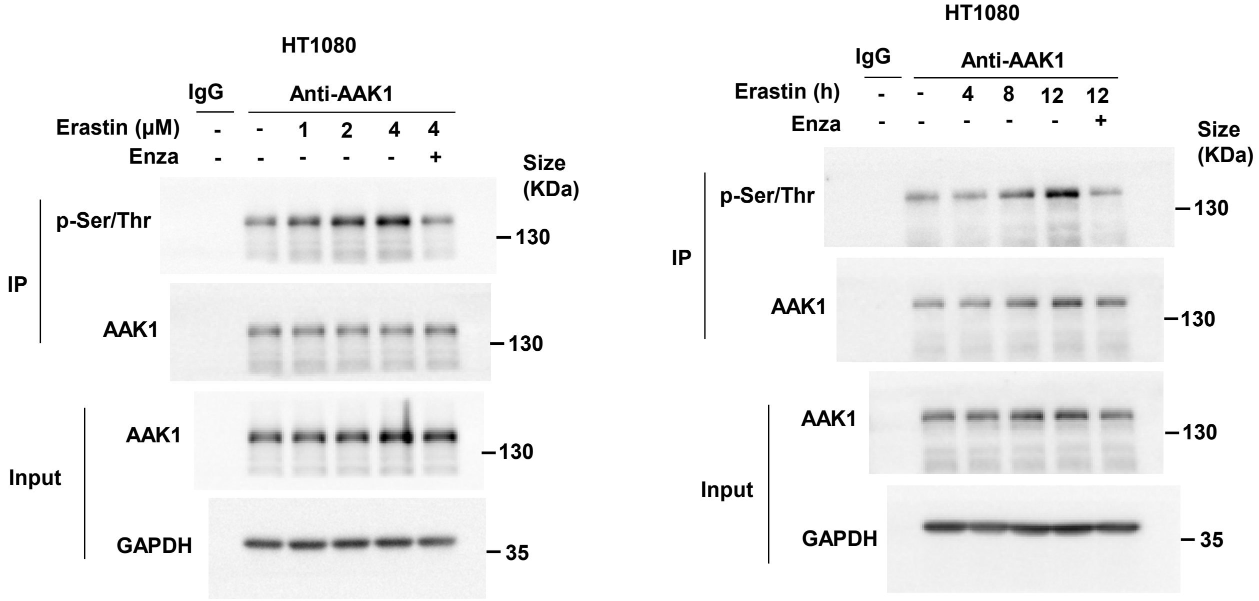

Supplementary Fig. 5C

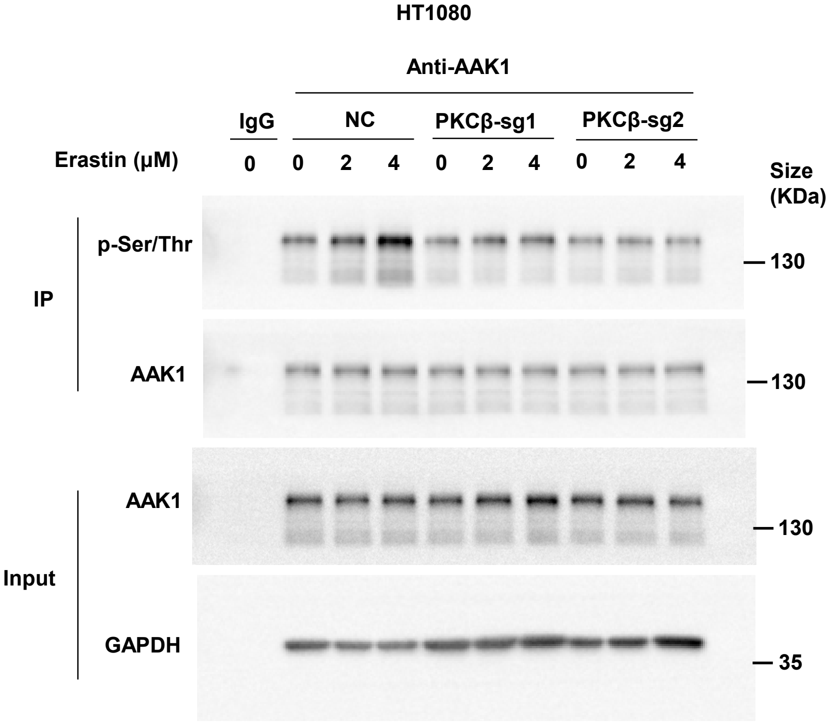

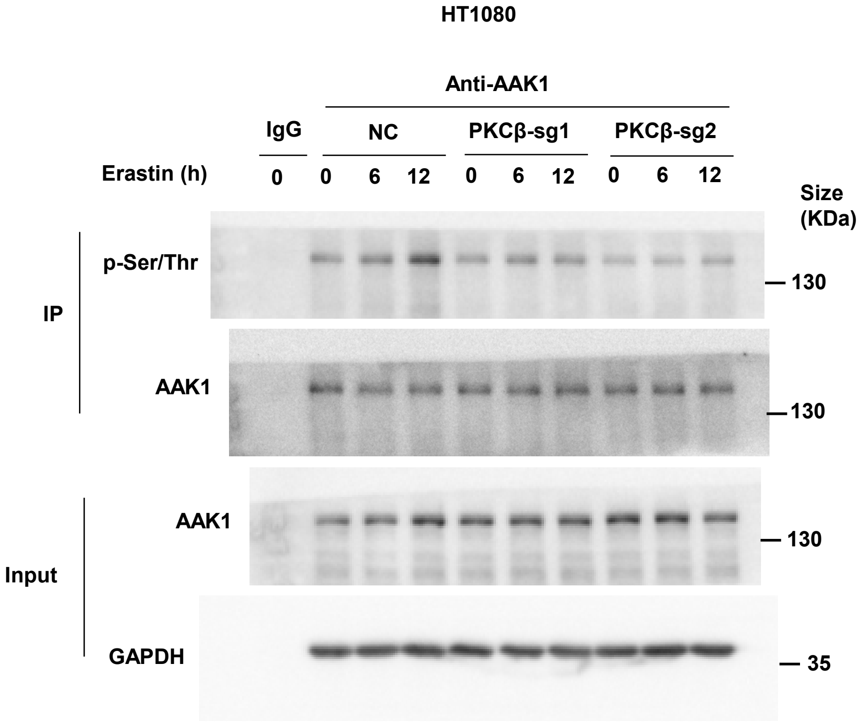

Supplementary Fig. 5D

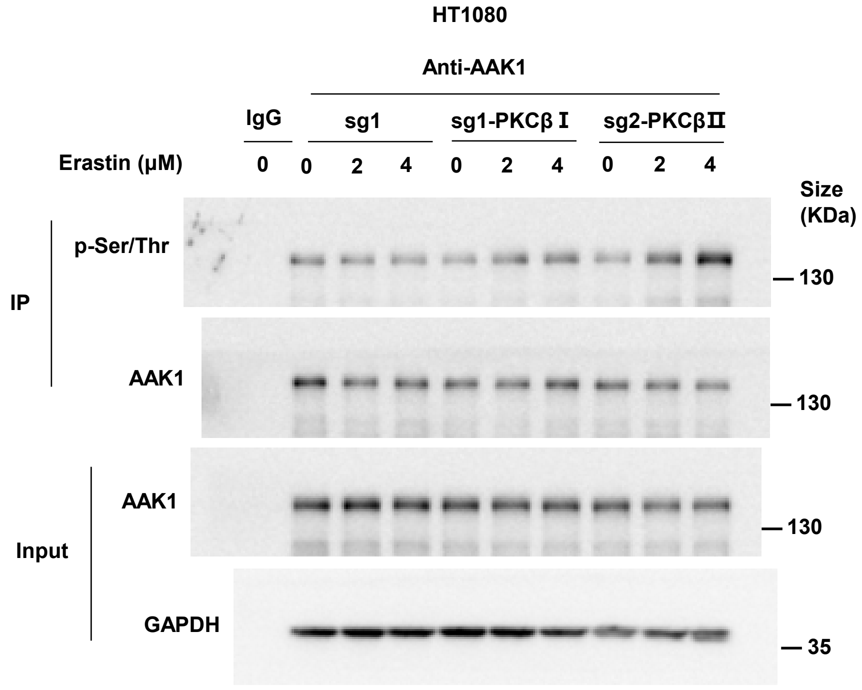

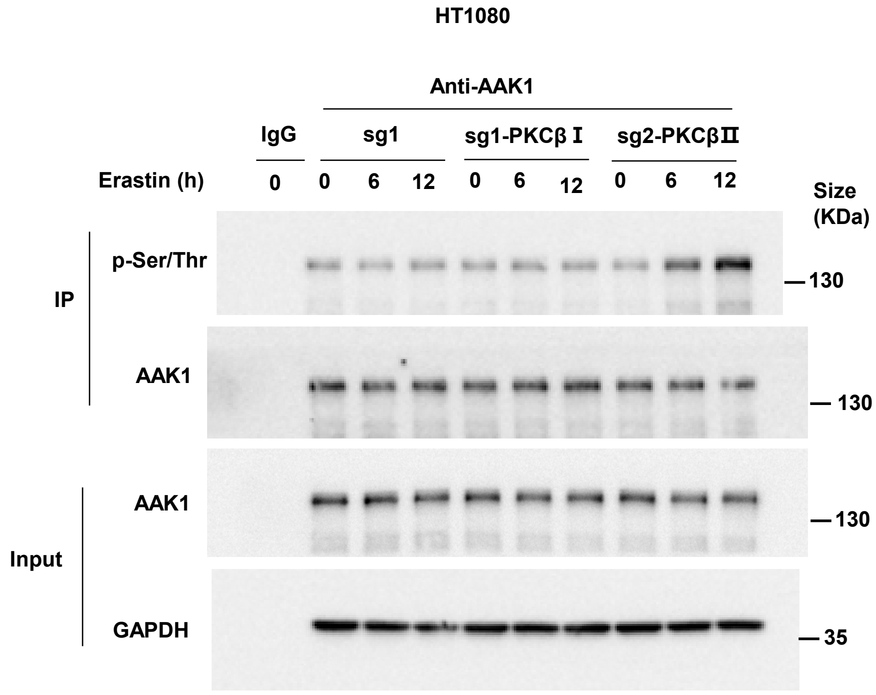

Supplementary Fig. 5E

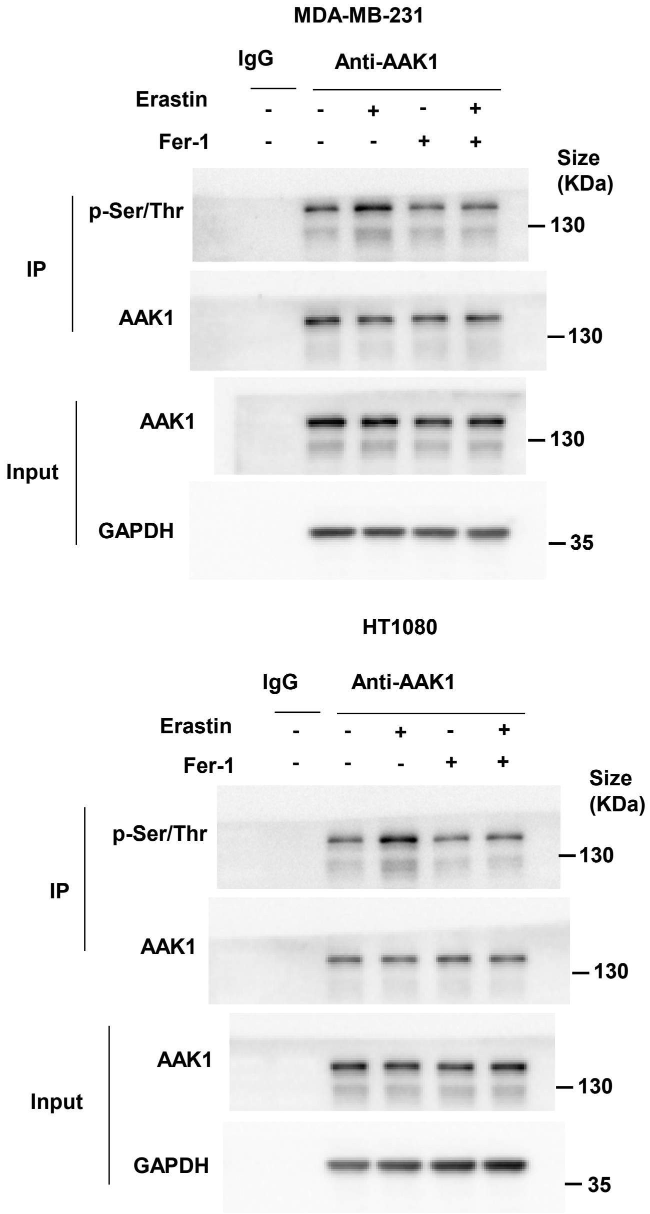

Supplementary Fig. 5G

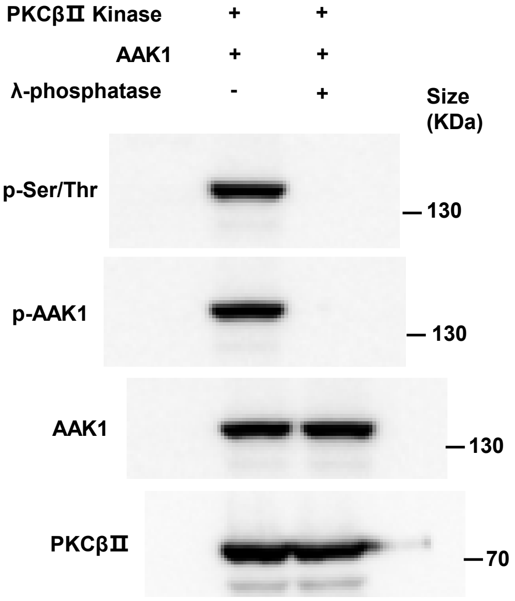

Supplementary Fig. 5F

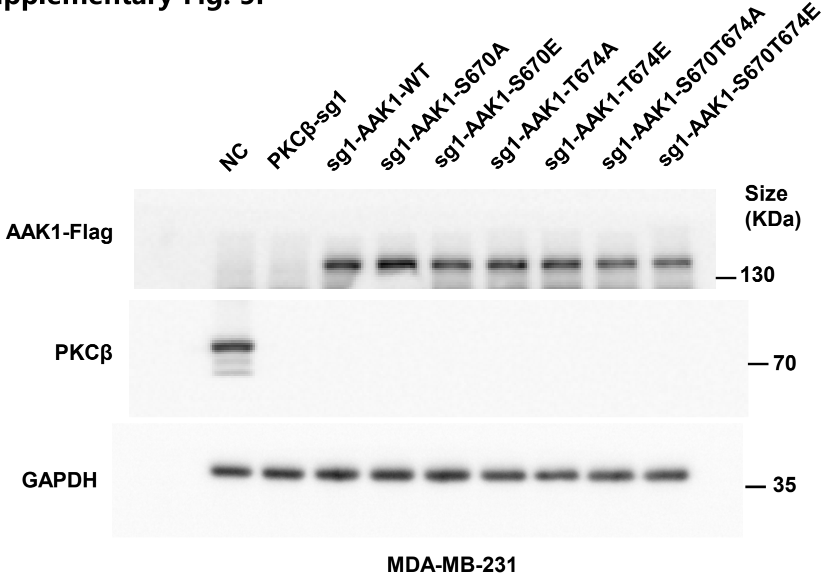

Supplementary Fig. 6A

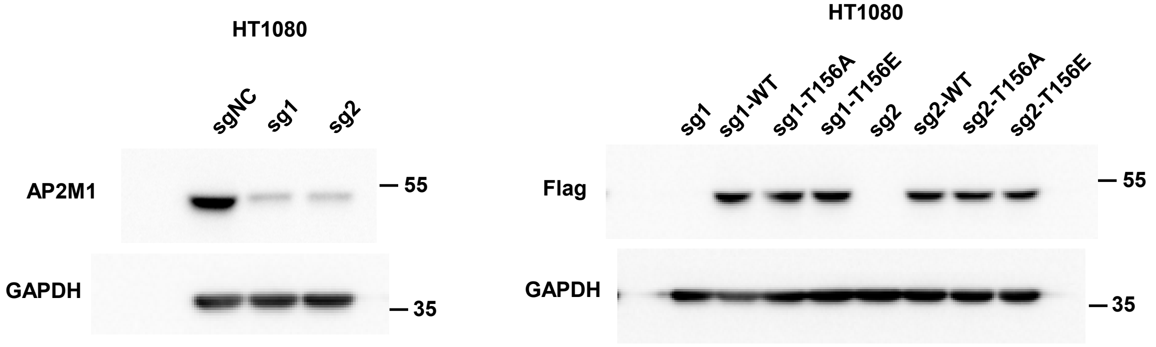

Supplementary Fig. 6C

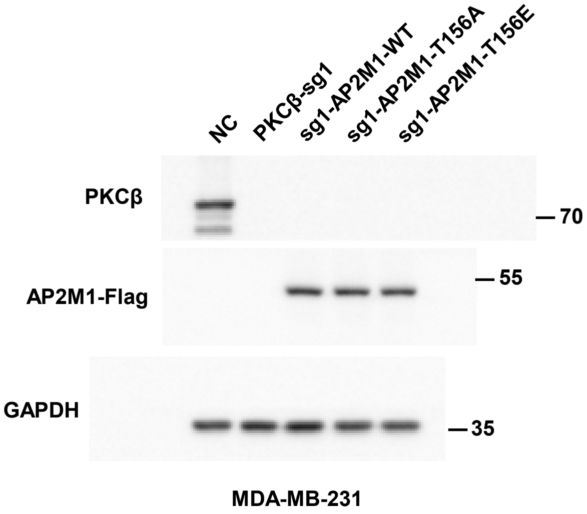

Supplementary Fig. 6D

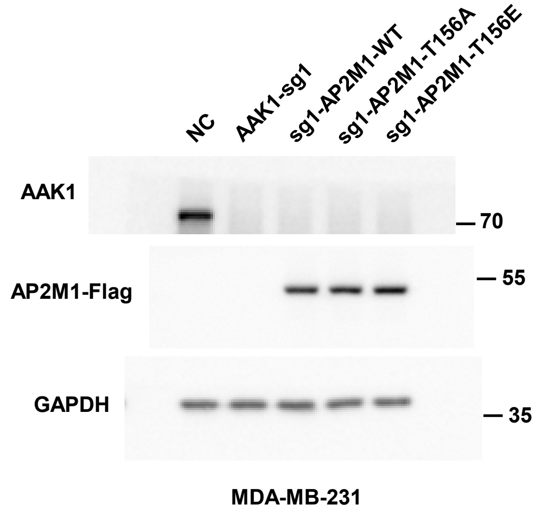

Supplement: Supplementary file 6 — Source Data (Blots and Gels) [file 41467_2025_67523_MOESM6_ESM.pdf]
